# Exponential Signaling Gain at the Receptor Level Enhances Signal-to-Noise Ratio in Bacterial Chemotaxis

**DOI:** 10.1371/journal.pone.0087815

**Published:** 2014-04-15

**Authors:** Silke Neumann, Linda Løvdok, Kajetan Bentele, Johannes Meisig, Ekkehard Ullner, Ferencz S. Paldy, Victor Sourjik, Markus Kollmann

**Affiliations:** 1 Zentrum für Molekulare Biologie der Universität Heidelberg, DKFZ-ZMBH Alliance, Heidelberg, Germany; 2 Institute for Theoretical Biology, Humboldt Universität zu Berlin, Berlin, Germany; 3 Department of Physics and Institute for Complex Systems and Mathematical Biology (ICSMB), Aberdeen, United Kingdom; 4 Department Biologie, Heinrich-Heine-Universität, Düsseldorf, Düsseldorf, Germany; Northeast Agricultural University, China

## Abstract

Cellular signaling systems show astonishing precision in their response to external stimuli despite strong fluctuations in the molecular components that determine pathway activity. To control the effects of noise on signaling most efficiently, living cells employ compensatory mechanisms that reach from simple negative feedback loops to robustly designed signaling architectures. Here, we report on a novel control mechanism that allows living cells to keep precision in their signaling characteristics – stationary pathway output, response amplitude, and relaxation time – in the presence of strong intracellular perturbations. The concept relies on the surprising fact that for systems showing perfect adaptation an exponential signal amplification at the receptor level suffices to eliminate slowly varying multiplicative noise. To show this mechanism at work in living systems, we quantified the response dynamics of the *E. coli* chemotaxis network after genetically perturbing the information flux between upstream and downstream signaling components. We give strong evidence that this signaling system results in dynamic invariance of the activated response regulator against multiplicative intracellular noise. We further demonstrate that for environmental conditions, for which precision in chemosensing is crucial, the invariant response behavior results in highest chemotactic efficiency. Our results resolve several puzzling features of the chemotaxis pathway that are widely conserved across prokaryotes but so far could not be attributed any functional role.

## Introduction

Information processing in living cells is limited by a complex balance between randomizing and correcting intracellular forces [1]. In particular, the stochasticity of biochemical reactions leads to fluctuations in the abundance and activity of cellular components, including those involved in cellular signaling. Although rapid fluctuations within a signaling cascade – arising from conformational changes, phosphorylation and binding events – are in most cases filtered by the comparatively slow phenotypic response of the cell, fluctuations on slower time scale can strongly affect cell’s precision to adapt to changing environmental conditions. Consequently, stochastic bursts in protein synthesis can interfere with the response to extracellular stimuli, making noise in gene expression one of the dominant noise sources that produce significant cell-to-cell variation in the response behavior of clonal cells [2]. The canonical way to reduce molecular noise is to increase copy numbers of genes, mRNA, and protein and to optimize their associated turnover rates [3]. The obvious disadvantage is that these solutions are of high cost to the cell and it is therefore not surprising that unicellular organisms employ more resource efficient strategies to control intracellular noise. Significant research efforts have been devoted in the last years to understand noise compensatory strategies of cellular circuits and to identify the underlying mathematical principles [1], [4]–[8].

However, most of the previous work focused on the consequences of cellular noise on the stationary pathway output [9]. To what extent cellular systems manage to eliminate effects of noise on response amplitude and relaxation dynamics is yet unclear. In the following we investigate signaling pathways that show precise adaptation – that is the relaxation of the output signal to its pre-stimulated level, even when the changed input persists. Adaptation can be realized by integral feedback or feedforward control [10] and allows living cells to ensure homeostasis of reaction fluxes and component concentrations, to expand the input range of molecular sensing devices, and to adjust the pre-stimulus activities of signaling cascades to the level of highest pathway sensitivity [11]. Well studied molecular circuits, where the existence of integral feedback loops have been experimentally confirmed, are the chemotaxis pathway in *E. coli* [12] and the hyperosmotic shock-response system [13] in *S. cerevisiae*. For these and most other cellular signaling systems there exists a strict time scale separation between rapid signal transduction from sensory molecules to the pathway output and comparatively slow changes of the dominant noise sources. Examples of the latter are stochasticity in synthesis and degradation of pathway components, assembly of large protein complexes, and changes in availability of cellular resources, such as ribosomes and RNA polymerases. These noise sources are in general multiplicative, show large standard deviations, and therefore do not allow description by linear noise approximation [3]. In this work we introduce a novel noise compensatory mechanism that allows to eliminate effects of multiplicative noise on signaling amplitude and response time for systems that show precise adaptation.

## Results

**Figure 1:**
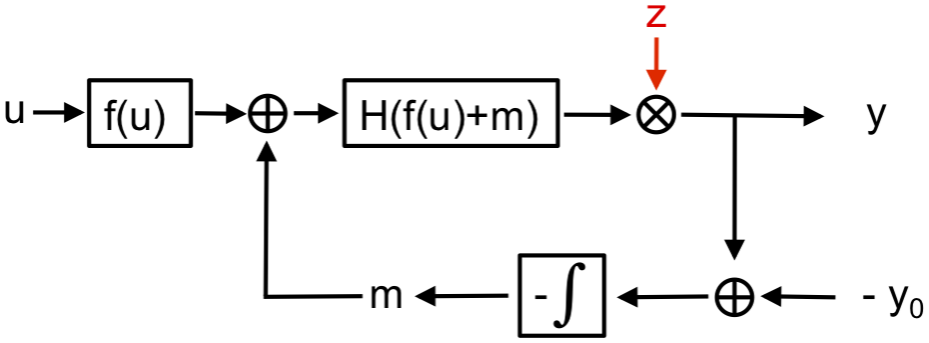
Signaling system with input 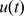 and gain function 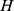. The output, 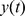, is controlled by an integral feedback loop, employing an auxiliary component 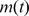, and approaches the adapted state 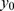 for 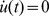. Multiplicative noise, with correlation times much longer than the relaxation time of the integral feedback, enters the system either via 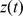 affecting both 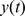 and 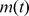.

**Figure 2:**
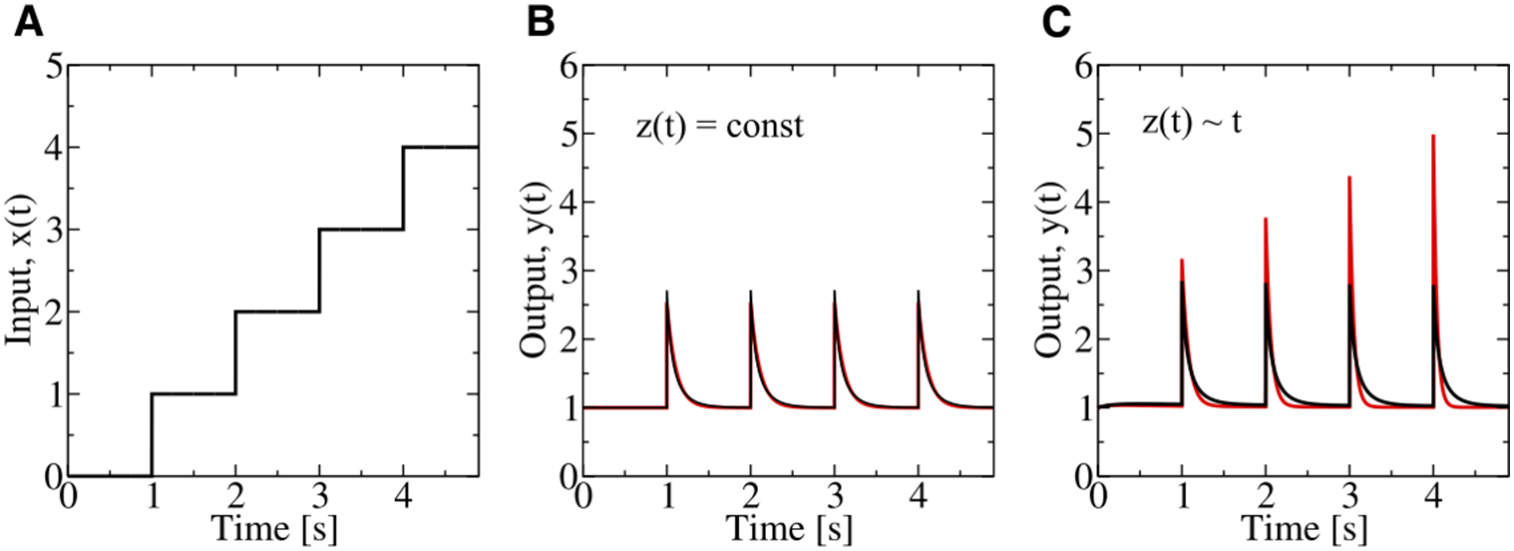
Response behavior of the system shown in Fig. 1 and described by Eq. (3). (A) Stepwise increase of the input function 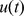 over time 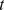. (B) Response dynamics of the output signal, 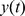, in absence of noise 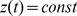, for 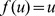 and two different gain functions 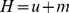 (red line) and 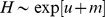 (black line). (C) Same as (B) but for slowly varying noise, simulated by a linear increase 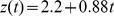. Here, the choice 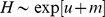 (black line) ensures robust response dynamics, in contrast to 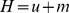 (red line).

The flow chart of Fig. 1 shows a simple example of adaptive signaling systems subject to slowly varying multiplicative noise 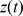. Exact adaptation can be achieved by an integral feedback, mediated by an intermediate variable 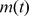. The dynamical system shown in Fig. 1 can be described by the equations
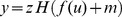
(1)

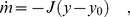
(2)where 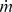 denotes the time derivative. The monotone functions 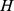 and 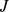 determine signaling gain and adaptation kinetics, respectively, with 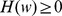 for all real values 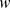 and 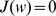 for 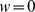. These functional constraints on 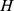 and 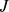 ensure that the pathway output, 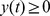, always relaxes to the adapted state, 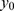, for 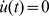. The equations above can be written as a single equation
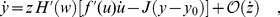
(3)using the definition 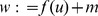. Here, 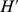 and 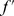 denote the derivatives of 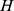 and 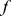, respectively. If the characteristic time scales of changes in 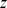 are significantly longer than the adaptation dynamics – as it is typically the case – the contribution of order 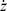, denoted by 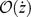, can be neglected to good approximation. For the same reason any slowly varying additive noise can be neglected in Eq. (1). Given a stepwise increasing input function, 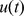, (Fig. 2A) and a linear signaling gain, 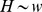, the adaptation dynamics of the output 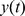 is identical for each step for 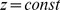 (Fig. 2B, red line). To mimic slowly varying multiplicative noise we introduce by 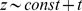 a perturbation that increases linear in time. This perturbation affects both response amplitude and relaxation dynamics of the signaling system (Fig. 2C, red line).

An unexpected change in the response dynamics occurs if the gain function satisfies the differential equation 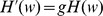, with 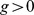. As a consequence, 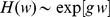 and Eq. (3) changes to
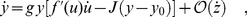
(4)which has the profound effect that the noise term is eliminated and the system has become dynamically robust against 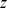. This case is illustrated in Fig. 2C (black line), where a dynamically changing function 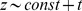 results in the same response behavior as for 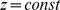, (Fig. 2B, black line). Thus, the effect of noise on amplitude and relaxation dynamics is eliminated simultaneously. This rather surprising result follows from the existence of a *symmetry* property of dynamical systems that show exact adaptation. Here, symmetry refers to invariance of the dynamic behavior under a continuous change of at least one system parameter. To illustrate this point we introduce a generic two-variable adaptation system,



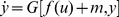
(5)

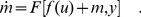
(6)


**Figure 3:**
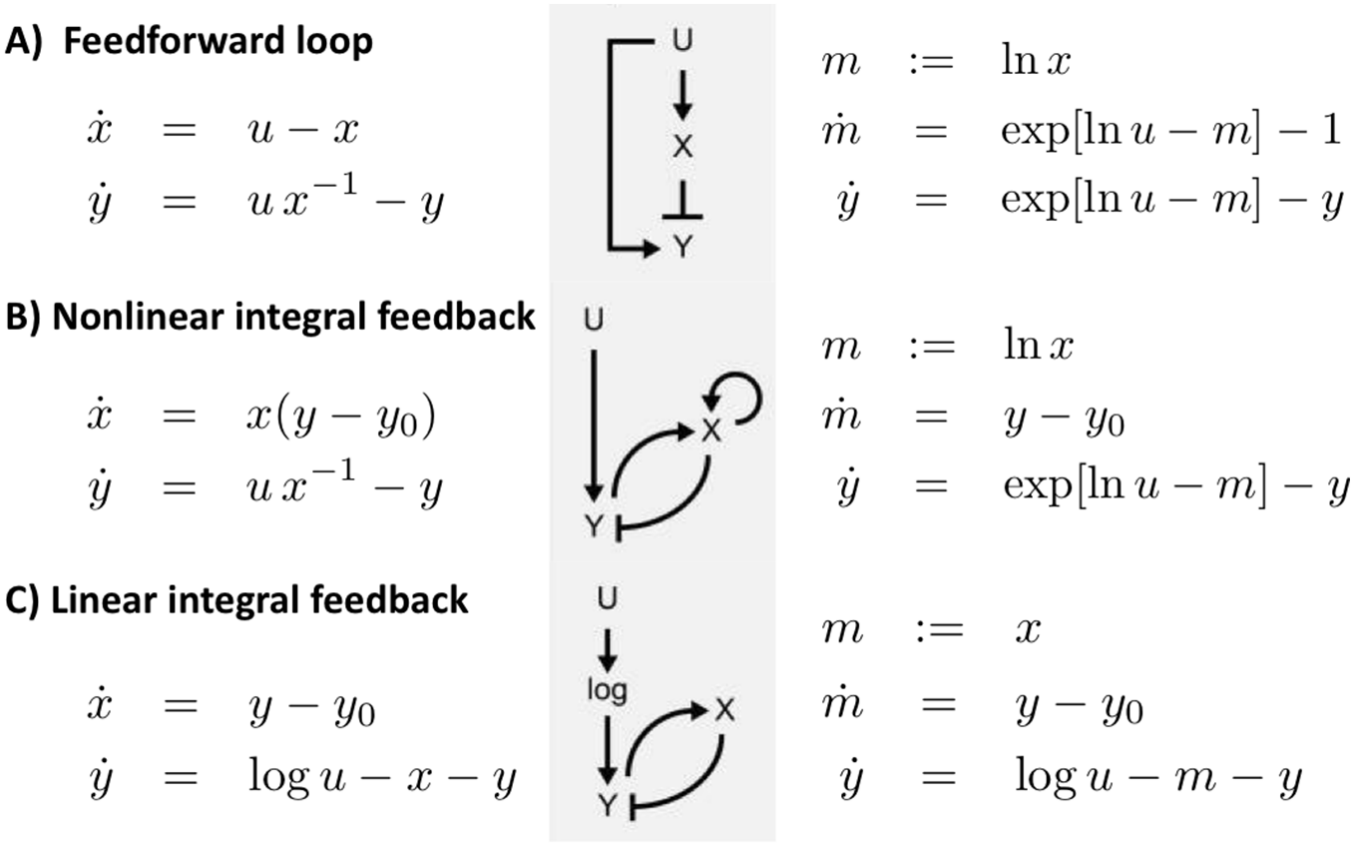
Network motifs that show dynamic invariance against rescaling of the input. Rescaling is defined by 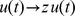, with a constant factor 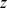. (A) Incoherent feedforward loop, (B) nonlinear integral feedback, and (C) linear integral feedback. Suitable transformation of the intermediate state variable, 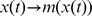, transforms the adaptation networks to the functional form shown in Eqs. (5) and (6), confirming the required logarithmic input dependency as explained in the main text.

The functions 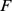 and 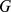 are monotone in both arguments and chosen such that the stationary output adapts to the stable fixpoint 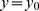 for all initial conditions. Note that Eqs. (5) and (6) do not represent the most general form of a two-variable adaptation system [14], as a generalization is possible by the substitutions 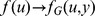 in Eq. (5) and 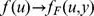 in Eq. (6), such that 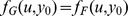. Frequently studied cases of Eqs. (5) and (6) are linear feedback loops, 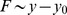, and linear feedforward loops, 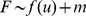. It is important to note that the functional forms of Eqs. (5) and (6) reflect the general principle of exact adaptation. This principle requires the existence of an intermediate variable 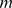 that relaxes under steady-state conditions, 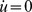, to 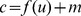, with 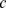 a fixed constant that is uniquely determined by 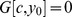. As a consequence, any transformation 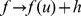, with 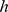 a constant, implies 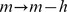 after a transient relaxation time. In other words, the system looses any information about the constant offset 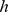 on longer time scales. As a consequence, the adaptation system, Eqs. (5) and (6), is dynamically invariant against a constant perturbation 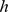 of the form 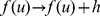 and dynamically invariant up to order 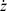 if 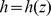 is a slowly varying function on the time scale of adaptation. A simple application of this concept is fold-change detection, which has been recently reported [14]. Here, the authors search for adaptation systems that show invariant output dynamics for the transformation 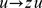, with 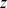 a constant scaling parameter. From our analysis it is clear that in this case the existence of invariant output dynamics requires input signals of the form 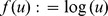 to result in the desired linear combination of signal and scaling parameter, 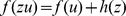, with 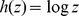. Using the illustrative examples of Shoval *et al.*, the associated dynamically invariant equations can be easily identified (Fig. 3). Our approach generalizes the results of Shoval *et al.* in the sense that we seek to transform a noisy, dynamic system to the functional form shown in Eqs. (5) and (6) by choosing appropriate functions 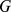, 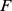, 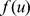, and 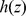, such that the perturbation 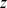 enters the system exclusively via the transformation 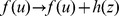.

### Biochemical Constraints

The question arises, whether living systems make use of the aforementioned *symmetry* property to eliminate slowly varying perturbations from biochemical circuits. Returning to the generic signaling network, Eq. (4), we first ask whether the condition 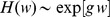 is a biochemically acceptable constraint. To this end we assume that the integral feedback loop is realized by changing the free energy, 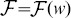, of fast equilibrating conformational states, 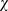, of a receptor protein. Assuming the existence of only two such conformational states – the 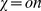 state that activates and the 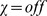 state that inactivates the pathway output – the probability to find the receptor in an active state is given by 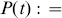 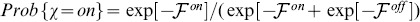, with the state dependent free energies, 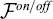, given in units of 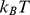, with 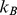 the Boltzmann constant and 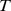 the temperature. If the free energy difference 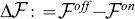 is determined by 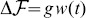 in the physiological relevant regime, we can identify the gain function as 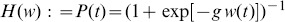. Assuming further that the equilibrium is dominated by the inactive state, 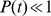, we obtain 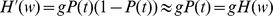. Thus, 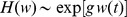 holds to good approximation within a certain regime of low receptor activity, which is determined by the conformational free energies of receptor states. What remains to be shown is that this concept has been realized in living systems.

### The Bacterial Chemosensory Pathway as Model System

**Figure 4:**
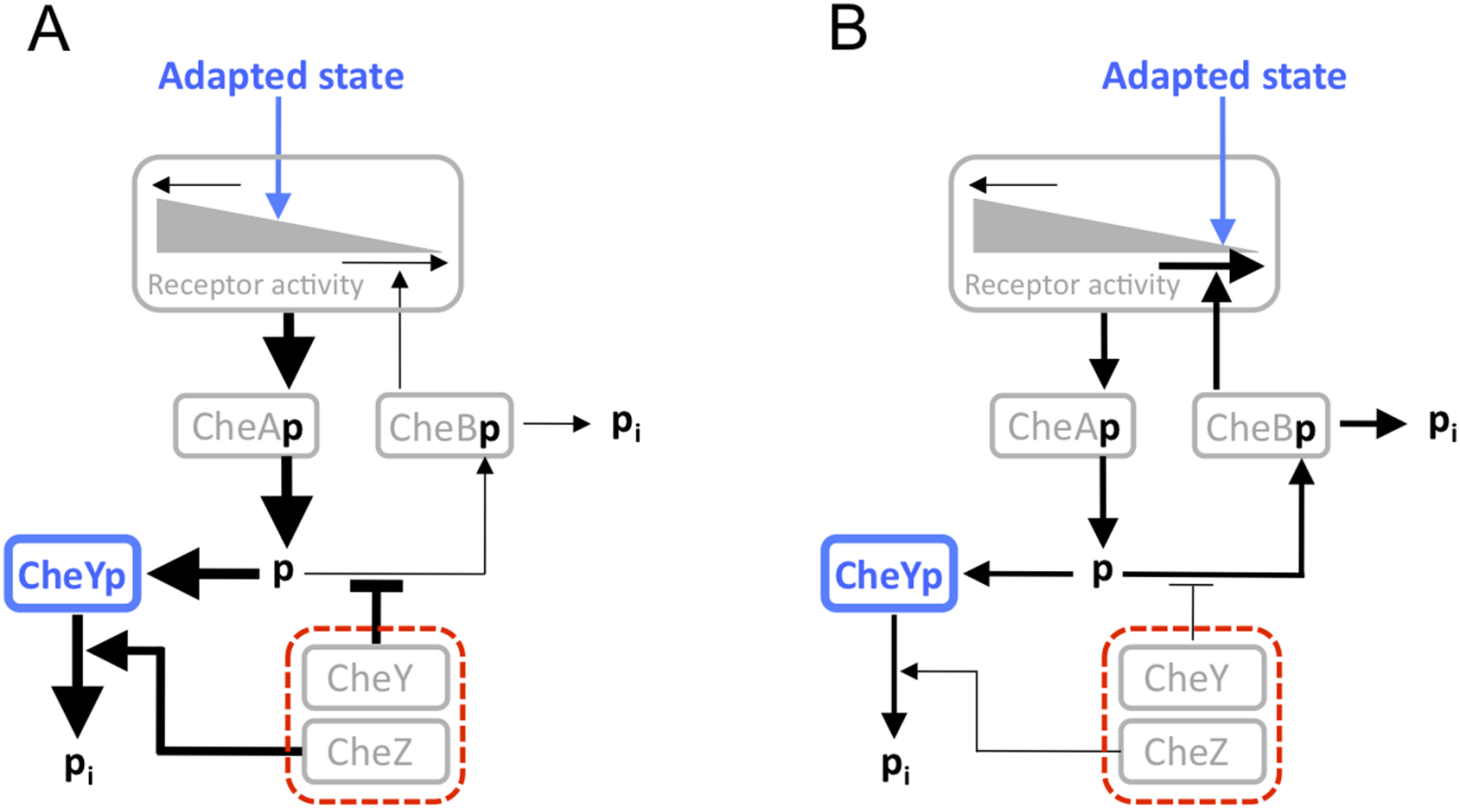
Cartoon of the phosphoflux regulation of the *E. coli* chemotaxis pathway. CheBp and CheYp indicate the phosphorylated forms of CheB and CheY, respectively. The adapted receptor activity is determined by the ratio between the methyltransferase flux – determined by the concentration of the methytransferase CheR (not shown) – and the methylesterase flux – determined by the concentration of the methylesterase CheB, whose activity is strongly increased upon phosphorylation. (A) Concerted upregulation of CheY and CheZ results in the perturbation 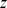 shown in Fig. 1. Upregulation of the major phosphate sink CheY relative to CheB decreases methylesterase activity, resulting in an increased receptor activity. Higher receptor activity in turn compensates the increased phosphatase activity of CheZ and results in an invariant pathway output, CheYp. (B) The inverse scenario takes place upon concerted downregulation of CheY and CheZ. As only concentration ratios matter, concerted downregulation of CheY and CheZ has the same effect as concerted upregulation of the remaining pathway proteins, CheRBAW and chemoreceptors.

A particularly suitable cellular system for such analysis, whose response dynamics requires high precision under intracellular noise, is the *E. coli* chemotaxis pathway (Fig. 4). This pathway has evolved to navigate bacteria in gradients towards favorable environments and shows outstanding sensitivity for changes in chemoeffectors, such as amino acids. Bacterial chemotaxis relies on temporal comparisons of concentrations of attractants or repellents along the swimming track of the cell [15]. A swimming trajectory consists of straight swim runs (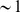
sec) interrupted by fast changes in orientation (tumbling events, 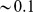
sec). Swimming in direction of favorable environments prolongs the swim duration, whereas swimming in opposite direction shortens it. Chemotactic stimuli are detected by stable sensory complexes consisting of receptors that are coupled to a kinase CheA with the help of an adaptor protein CheW, whereby binding of attractants downregulates the autophosphorylation activity of CheA and binding of repellents has the opposite effect. These sensory complexes are organized in large clusters, where allosteric interactions between receptors serve to largely amplify the signals on the level of kinase control. Phosphorylated CheA rapidly transfers its phosphoryl group to the response regulator CheY, which binds to flagellar motors and causes them to switch from the counterclockwise (CCW) to clockwise (CW) direction of rotation. The pathway further includes a CheY phosphatase, CheZ, and an adaptation system that consists of two receptor modification enzymes, the methyltransferase CheR and the methylesterase CheB. The pathway activity shows almost perfect adaptation under constant stimulation [12], [15]. This adaptive behavior is the result of an integral feedback from the sensory complex activity to the activity of the adaptation system, mediated at two levels: enzyme specificity of CheR/CheB for inactive/active receptors and CheB phosphorylation. Furthermore, the *E. coli* chemotaxis pathway shows an exceptionally high signaling gain [16] and mechanisms to compensate for the detrimental effects of gene expression noise on the adapted state [6], [17]. These properties indicate the existence of a high selection pressure to maximize the signal-to-noise ratio in detecting changes of chemoattractants and suggest that further noise compensatory mechanisms might have evolved to ensure precision of response amplitude and adaptation dynamics under intracellular perturbations. Although other noise sources such as fluctuations in the assembly of fully functional receptor clusters might affect the chemotaxis behavior, we focus in this work exclusively on the effects of gene expression noise, which is one of dominating noise sources in bacterial signaling [6].

### Mathematical Modeling of Chemoattractant Response Kinetics

We now introduce a simplified mathematical model that describes the response dynamics of the chemotaxis pathway in vicinity of the adapted state with sufficient accuracy. Due to simplicity, this mathematical model cannot capture the complete response behavior of the chemotaxis pathway, such as nonlinear behavior in the methylation dynamics with respect to receptor activity [19]. To describe receptor activation we employ a highly accurate model of cooperative chemoreceptor interactions in *E. coli* as introduced previously [20], [21]. In this model, a number of 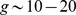 receptor homodimers form an allosteric complex and both the specific chemoeffector concentration, 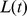, and the average methylation level, 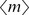, can change the free energy difference 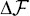. The free energy difference determines the probability of allosteric receptor complexes in be in an active state
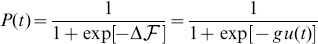
(7)Here, 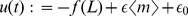, with 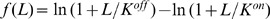. The constant 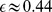 has been determined using a linear regression model for the experimentally measured kinase activities and 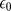 represents an offset value. Dissociation constants for chemoeffectors in the ‘on’ and ‘off’ state are denoted by 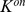 and 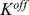, respectively, with 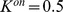
mM and 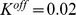
mM for methylaspartate [16], [20], [21]. The monotone increasing function 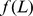 is the average free energy increase per receptor due to ligand binding. We further approximated the mean methylation level of the individual allosteric receptor complexes by the average methylation level of receptor homodimers, 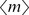. Previous results [20] justify these approximations for 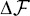: the additivity between the free energy contributions arising from both ligand binding and receptor methylation and the linear dependence of the free energy on the mean receptor methylation level. Recent experiments [19] further showed that in the regime of 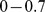 the maximum receptor activity the dynamics of 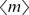 can be described to leading order by the rate equation
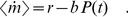
(8)Here, 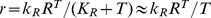 and 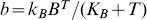 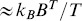 are determined by the total concentrations of CheR, 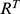, CheB, 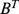, and chemoreceptors, 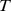, with 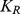 and 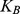 the associated Michaelis-Menten constants. As both chemoreceptors and CheR/CheB are part of the same regulon, the ratio between 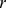 and 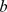 shows only small intrinsic noise [6] and can be treated as constant. From Eq. (8) follows that the adapted receptor activity, 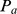, is determined by
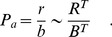
(9)Taking the time derivative of Eq. (7) results in the expression
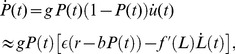
(10)with 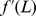 the derivative of 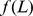. The second line of Eq. (10) assumes that 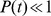 in vicinity of the adapted regime. This assumption will be justified experimentally in the following and allows us to neglect differences in the methylation rate of CheR when bound to active or inactive receptors.

The reason why it makes sense for the cell to keep only a small proportion of receptors active in the adapted state is that the sensitivity to stimuli is maximal in limit 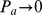. This can be seen by calculating the signaling gain, 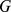, which is defined by the relation 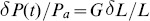, with 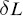 a sufficiently small change in ligand concentration and 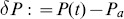. From Eq. (7) we obtain 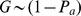, which confirms that the signaling gain is maximized in the limit 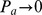. However, a small 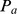 significantly reduces the dynamic range over which attractant can be detected as in this case 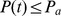. For example reducing the dynamic range from 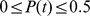 by ten-fold to 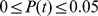 implies that a non-saturating response to chemoattractant results in ten-fold reduced signal-to-noise ratio at the receptor level. Although the correlation times of the conformational receptor states and ligand binding are short in comparison to the turnover of CheYp at the flagellar motor complex – which implies averaging over noise at the receptor level – there exists a tradeoff between increasing the signaling gain and reducing the signal-to-ratio upon decreasing 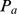. Additionally, lowering 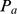 requires a simultaneous increase in kinase activity to reach the required level of phosphorylated CheY. In fact the kinase activity of CheA is orders of magnitude higher than the kinase activity of other two-component systems.

Following Fig. 1 we denote the physiological relevant signaling output by 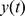. As 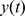 is proportional to the receptor activity, we introduce with 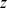 a stochastic proportionality factor, 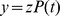, which is determined by the fluctuating amount of receptor kinase complexes relative the concentration of CheZ. Fluctuations in receptor kinase complexes affect both the output signal, 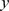, by changing the level of CheY phosphorylation and the feedback strength by changing the level of CheB phosphorylation. Consequently, the transformed differential equation for the signaling output is given by
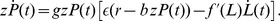
(11)Substitution of 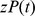 shows that relative fluctuations in kinase activity are eliminated by the same mechanism as presented in Eq. (4)
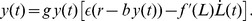
(12)For a step increase in chemoeffector concentration at time 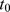, 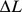, under otherwise constant conditions, the solution of Eq. (12) is given by
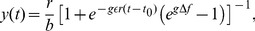
(13)with 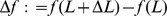. This result shows that the adaptation dynamics to step changes in chemoattractant is determined by the rate 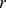 but independent of the rate 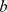.

### Experimental Confirmation of Dynamic Invariant Behavior in *E. coli*

**Figure 5:**
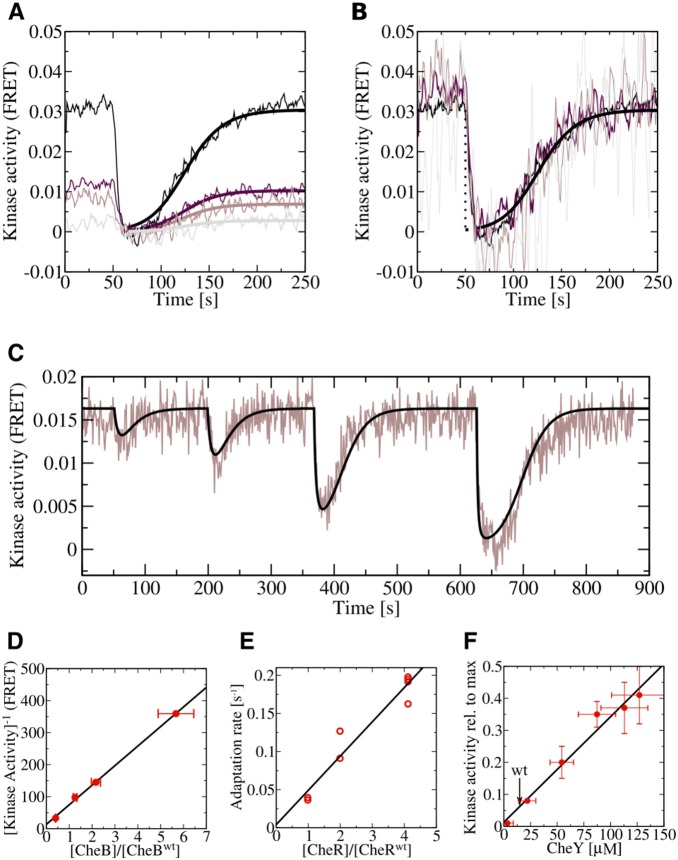
Scaling behavior of adaptation dynamics and of kinase activity. (A) FRET measurements of kinase activity upon sudden addition of attractant, 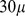
M 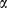-DL-methylaspartate, at 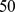
s. Expression of CheY-YFP/CheZ-CFP FRET pair was induced by 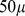
M IPTG. The FRET signal was recorded every second and smoothened by sliding window of four seconds. CheB was expressed from plasmid at varying levels (0% to 0.01%) of arabinose yielding 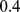-fold (black line), 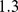-fold (dark brown line), 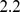-fold (light brown line), and 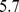-fold (gray line) the native expression level. Bold solid lines are the predictions of the mathematical model, Eq. (13), with adaptation rate 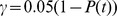. (B) Adaptation dynamics from (A) after rescaling of the kinase activity with the CheB expression level. (C) FRET measurements of kinase activity (brown line) and corresponding theoretical prediction (black line) for a sequence of increasing steps (1
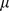
M, 3
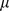
M, 10
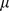
M, and 30
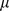
M) of 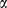-DL-methylaspartate. (D) Linear relationship between CheB expression level and adapted kinase activity. (E) Linear relationship between CheR expression level and the adaptation rate. (F) Linear dependency of kinase activity on concerted changes in CheY-YFP/CheZ-CFP expression level. Lines in the panels (D–F) show least-squares fits to the data and error bars denote standard errors.

In the following we give experimental evidence that *E. coli* cells operate in the regime where noise compensation by exponential can be realized. To confirm that the functional form of Eq. (13) provides a good representation of the adaptation dynamics in *E. coli* chemotaxis, we measured *in vivo* the activity of the chemoreceptor associated kinase CheA in response to a step increase in chemoattractant while varying the levels of CheB and CheR (Fig. 5A). The measurement was carried out using phosphorylation-dependent interaction of CheY fused to yellow fluorescent protein (CheY-YFP) with its phosphatase CheZ fused to cyan fluorescent protein (CheZ-CFP) as an intracellular reporter of the pathway activity, as described previously [16]. The amount of the complex formed by the two proteins, which is directly proportional to the intracellular kinase activity (see below), was determined using fluorescence resonance energy transfer (FRET). Although receptor activity gradually decreased upon increase in CheB concentration (Fig. 5A,D), the adaptation kinetics remained invariant. Multiplying both sides of Eq. (13) by a factor proportional to the concentration CheB, 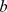, shows that 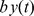 is independent of the concentration of CheB, which is confirmed by the collapse of the FRET response curves onto a single master curve (Fig. 5B). Moreover, our model could well reproduce the adaptation kinetics observed for attractant stimuli of increasing strength (Fig. 5C). On the other hand, the adaptation rate showed a linear dependence on the level of CheR, again consistent with Eq. (13). It would have been desirable to measure the response dynamics of attractant removal, as this would give additional experimental evidence that Eq. (13) is indeed correct in vicinity of the adapted state. However, there exists strong evidence for an abrupt change in the strength of the adaptation feedback in regime of high receptor activity [19], whose molecular origin has not yet been elucidated. Due to phenotypic heterogeneity of *E. coli* cells, a significant fraction of cells will enter the high activity regime upon attractant removal which make it hard if not impossible to infer from attractant removal the adaptation dynamics around the adapted state.

The phosphorylation rate of chemoreceptor complexes can be expected to be a product of 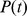, the CheA autophosphorylation rate, 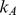, and the concentration of kinases CheA that are a part of functional receptor clusters, 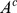, resulting in the kinase activity 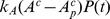, with 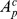 the amount of phosphorylated kinases CheA. Because of fast phosphate group transfer to CheY [22], almost all kinases are unphosphorylated near the adapted state, and the resulting kinase activity, 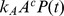, is balanced by the hydrolysis rate of phosphorylated CheY,
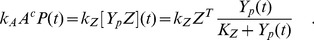
(14)Here, we denoted by 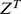 the total concentration of the phosphatase CheZ and by 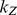 the associated dephosphorylation rate. The hydrolysis rate of CheYp is proportional to the concentration of CheZ/CheYp complexes, 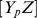, which are resolved by introducing the Michaelis-Menten constant 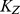 and the concentration of freely diffusible CheYp, 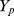, – the physiological relevant pathway output. To allow for direct comparison with Fig. 1, we define the signaling output, 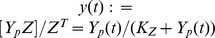, as a monotone increasing function of 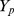. The signaling output can be directly measured by fluorescence resonance energy transfer (FRET) between CheY-YFP and CheZ-CFP, bicistronically expressed from a plasmid in a *cheY/cheZ* deleted strain [16], [23]. This technique detects the amount of CheY that is bound to CheZ up to a proportionality factor. As only phosphorylated CheY binds with significant affinity to CheZ, the light intensity emitted by the FRET pairs is proportional to 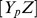. The background FRET signal can be determined using a saturating stimulus of chemoattractant, which switches off the receptors and thereby drives 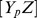 to zero. Measuring simultaneously the light intensity emitted by CheZ-CFP allows to infer the concentration 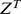, after calibration. The ratio between the FRET signal and the CheZ-CFP fluorescent signal scales linear with the signaling output
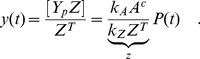
(15)Here, we used Eq. (14) to substitute the expression 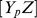 and defined the pathway perturbation, 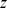, as the imbalance between the concentrations of functional receptor-kinase complexes and phosphatase CheZ.

Under physiological conditions, the stochastic properties of 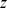 are likely dominated by fluctuations in the assembly of functional receptor-kinase complexes, 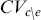, and intrinsic gene expression noise among the chemotaxis operons. The noise contribution arising from unequal distribution of receptor clusters among daughter cells is attenuated by the fact that a large fraction of CheZ stably associates with receptor clusters – thus preserving the 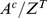 ratio [24].

As variation in the adapted pathway output has a negative effect on the chemotaxis efficiency, we wondered whether the *E. coli* chemotaxis system uses the concept of noise elimination by exponential signaling gain to preserve the adapted pathway output, the response amplitude, and the adaptation time in presence of large perturbations. Previously we have shown that an approximately 7-fold upregulation of all proteins involved in chemotaxis signaling – CheRBYZA and chemoreceptors – results in the same adapted pathway output as observed for the wild type system [6]. This outstanding degree of robustness could be attributed to a concerted concentration change of receptor-kinase complexes and phosphatase, leading to balanced phosphorylation and dephosphorylation of the response regulator CheY. Here, we perturb the phosphorylation flux balance of the chemotaxis pathway by strongly varying the expression level of CheY and CheZ, as schematically indicated in Fig. (4). This perturbation simulates the physiologically relevant imbalance between the fluctuating amount of fully functional receptor complexes and the downstream signaling proteins CheY and CheZ.

**Figure 6:**
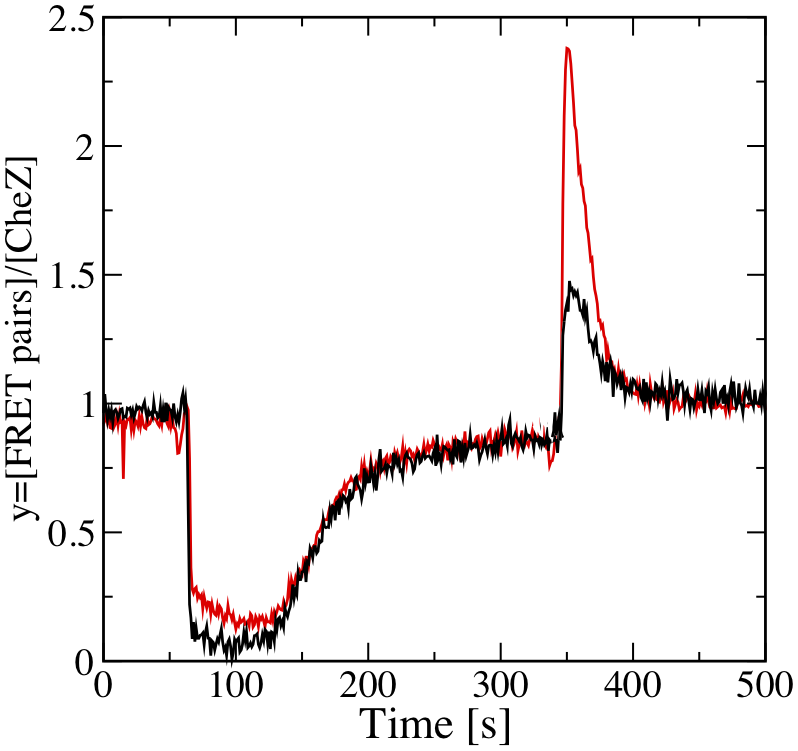
Experimental confirmation of dynamically invariant response behavior of the chemotaxis pathway in *E. coli* upon a perturbation indicated by 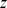 in Fig. 4. Shown are the effects of a decreasing ratio between the concentrations of CheY-YFP and CheZ-CFP and the remaining signaling proteins by 2-fold (red line) and 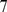-fold (black line), whereby CheY-YFP/CheZ-CFP were expressed in LL4 cells at 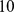 or 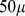
M IPTG induction, respectively. Cells were stimulated by addition and subsequent removal of 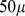
M MeAsp. The y-axis shows the normalized change in FRET signal, which is to good approximation proportional to the signaling output, 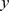, as explained in the main text. The base line of zero activity was determined by addition of saturating amounts of attractant.

Fig. 6 shows the response dynamics for a 7- and a 2-fold reduced concentration of the pathway proteins CheY and CheZ in comparison to the remaining proteins involved in chemotaxis signaling. Such variation was achieved by deleting the genes for CheY and CheZ from the genome and expressing them from plasmid as fluorescent fusion constructs, CheY-YFP and CheZ-CFP. From Eq. (15) it is clear that fold changes in the concentration of CheZ strongly reduces the signaling output, 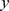, and thus the concentration of phosphorylated CheY. Surprisingly, no significant difference in the signaling output is observed upon elevating CheZ, while leaving the kinase activity unchanged (Fig. 6). This observation points to a regulatory mechanism within the chemotaxis system that can detect an imbalance between kinase activity and the concentration of CheZ, as their ratio determines the signaling output. The significant difference in the response amplitude upon attractant removal might be attributed to the saturating effects of the enzyme CheZ or the phosphoreceiver CheY, if both proteins are expressed at low level. As high precision of the chemotaxis signaling system in *E. coli* is mainly required for detecting shallow nutrient gradients, the adapted signaling output and the adaptation time are expected to be under strong selection, but not necessarily the response amplitude after strong negative stimulation. The observation of an unchanged response dynamics of the chemotaxis system despite a 7-fold reduction in phosphatase activity requires a molecular mechanism that regulates kinase activity proportional to the expression level of CheZ and CheY. The observed invariance in the response dynamics is also surprising from the evolutionary point of view, as a 7-fold difference in the concentration ratio between CheY/CheZ and the other pathway proteins seems to be far outside the physiological noise range.

Our first guess that the strong binding affinity of CheZ to the kinase CheA might be responsible for the observed kinase regulation turned out not to be true. We could reject this hypothesis by measuring the phosphotransfer rate from CheA to CheB for varying concentrations of CheZ in absence of CheY. The absence of any significant effect of CheZ concentration on kinase activity is shown in Table S1 in File S1. As there is no experimental evidence that kinase activity affects phosphatase activity [25], we are left with the remaining hypothesis that changes in CheY concentration affect kinase activity. This hypothesis is directly confirmed by FRET measurements (Fig. 5F) and by swarming experiments [26] where the observation of tumbling cells upon CheY overexpression suggests an increased concentration of CheYp and the observation of swimming cells upon CheZ overexpression indicates a decreased level of CheYp. In contrast, no significant change in tumbling frequency was observed upon concerted overexpresssion of CheY and CheZ, which is in agreement with the FRET results reported in this work.

Although the mechanism how CheY affects kinase activity remains to be investigated in more detail, we speculate that it might be at least partly related to the experimentally observed competitive binding between CheY and CheB to the phosphoreceiver domain of CheA [27]. This hypothesis is supported by the fact that the wild type concentration of CheY is around 30-fold higher than the concentration of CheB and that CheA autophosphorylation is the rate limiting step of phosphorylation of CheB and CheY. As phosphotransfer to CheY is the dominant phosphate sink in the system, the phosphotransfer rate to CheB is limited by the concentration ratio [CheB]/[CheY]. Consequently, the concentration of CheBp is to leading order inversely proportional to the concentration of CheY,
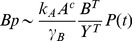
(16)with 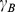 the autodephoshorylation rate of CheBp. Although it has been shown by *in vitro* and *in vivo* experiments that the methylesterase activity of CheB is enhanced several fold upon phosphorylation, it is generally believed that preferential demethylation of active receptors by CheB and preferential methylation of inactive receptors by CheR is the dominating mechanism of adaptation. However, most of our knowledge about the *in vivo* adaptation dynamics has been generated by FRET assays, with the CheY-YFP/CheZ-CFP FRET pair expressed almost one order of magnitude above the wild type level [16]. As shown in Fig. 5F this overexpression results in an order of magnitude elevation of the receptor activity and thereby strengthens the feedback via preferential methylation of active receptors. If CheY-YFP/CheZ-CFP is expressed at wild type level, the competition between CheY and CheB for phosphogroups at CheA shifts in favor for CheB phosphorlation and thereby strengthens the feedback via CheB phosphorylation. Under wild type conditions, the small proportion of active receptors do not significantly affect the net methylation rate of CheR and shuttling of phosphorylated CheB between the phosphoreceiver domain at CheA and the pentapeptide sequence at Tar and Tsr receptors might be sufficient to ensure that adaptation of receptor activity occurs locally. This scenario is further supported by the high autodephosphorylation rate of CheB that narrows the action range of phosphorylated CheB. Furthermore, the existence of a phosphorylated form of CheB seems to be under significant selection as it is an ubiquitous feature of bacterial chemotaxis although it is not required for adaption. Assuming that CheB phosphorylation dominates the adaptation feedback near the adapted state under wild type expression levels, we arrive at the expression
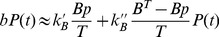
(17)with associated rate constants, 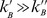. This functional form reproduces the experimentally observed linear dependence of the demethylation rate on receptor activity, 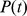, in the regime of low receptor acitivity [19]. As under wild type conditions the phosphorylated form of CheB seems to dominate the demethylation kinetics around the adapted state, we can neglect the contribution of non-phosphorylated CheB in Eq. (17) to describe the perturbation experiment, Figs. 4 and 6. Using the definition of 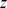 in Eq. (15), we can rewrite Eq. (17) as
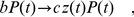
(18)where the constant 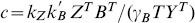 is invariant under concerted concentration changes of CheY and CheZ. Comparison with Eq. (11) shows that under these assumptions the chemotaxis signaling pathway is dynamically invariant against changes in 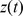.

Our analysis shows that competitive phosphorylation of CheY and CheB at CheA can serve to counterbalance any mismatch between kinase activity and the concentration of CheZ. The same mechanism can also compensate deviations from the optimal adapted state due to differences in expression levels between the individual chemotaxis operons. The compensatory mechanism relies on the fact that the concentration of CheY is an excellent proxy of the concentration of CheZ due to coexpression and translational coupling.

**Figure 7:**
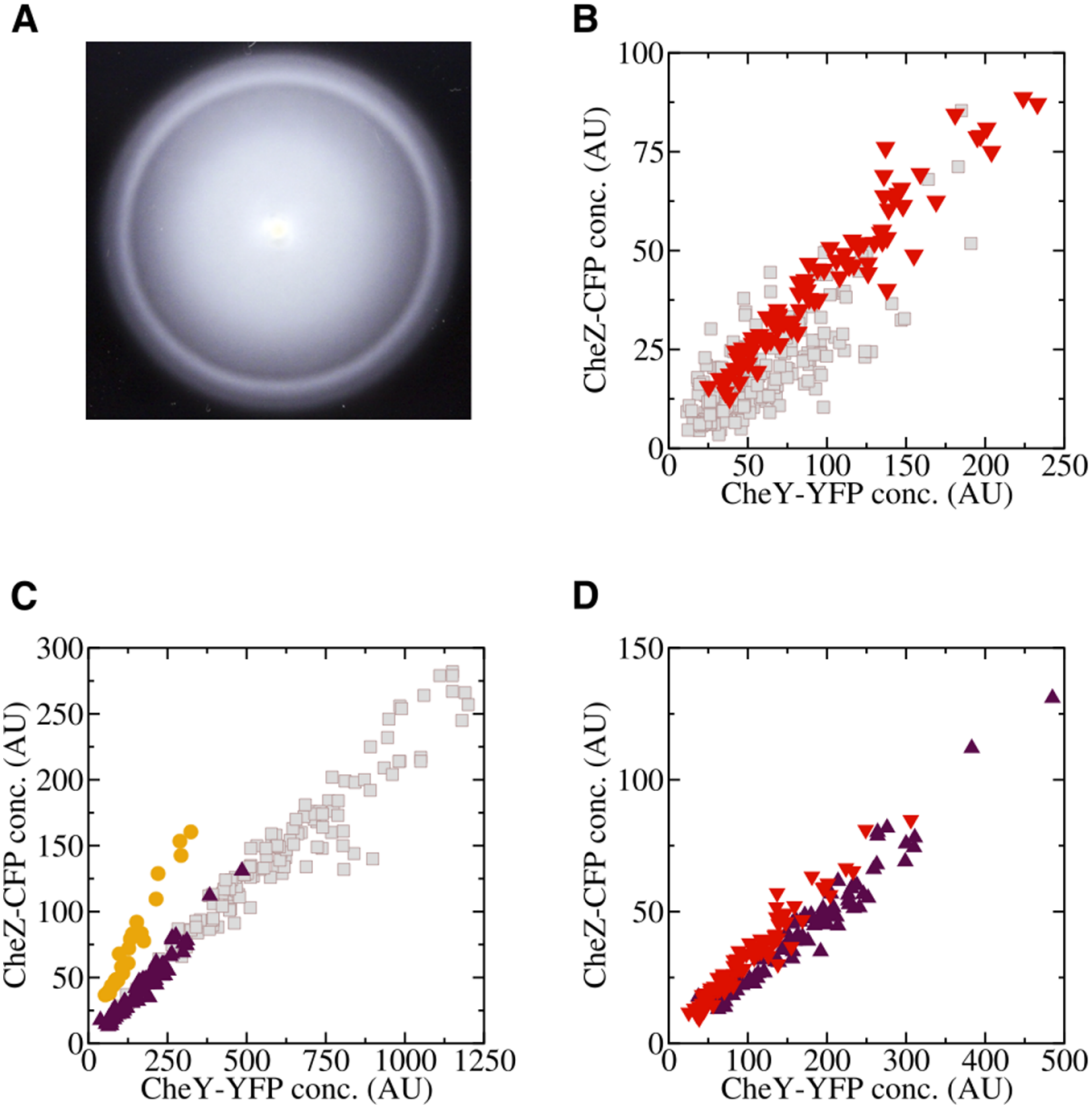
Phenotypic selection in chemotactic *E. coli* cells. (A) Picture of a chemotactic swarm ring produced by an *E. coli* population spreading for eight hours on soft agar. Cells with highest chemotaxis efficiency accumulate in the outer swarm ring. (B)-(D) Scatter plots of the concentrations of bicistronically expressed CheY-YFP and CheZ-CFP in individual cells taken from the inner (squares) and the outer (triangles and circles) swarm ring at different induction. Average expression levels in the culture were either below (0 IPTG), (B), or above (25 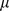
M IPTG), (C), the native level. Circles indicate cells expressing CheZ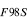-CFP that cannot bind to receptor clusters. Collapse of the scatter plots for the best swarming cells is shown in (D).

Our results suggest that strong coupling between the levels of CheY and CheZ is beneficial for optimal chemotaxis, as in this case CheY counterbalances CheZ activity by acting as a phosphate sink and thereby reduces the phosphotransfer rate to CheB. This can be directly demonstrated by selection for best-chemotactic cells on tryptone broth soft agar plates. On these plates, an attractant gradient is created at the edge of the growing colony by nutrient depletion due to cell growth and, as a consequence of chemotaxis, cells with highest chemotactic efficiency accumulate at the outer edge of the swarm ring [26] (Fig. 7A). We tracked cells by video microscopy to control that they were actively swimming. Indeed, when cells coexpressing CheY-YFP and CheZ-CFP in a *cheY/cheZ* deleted strain from a bicistronic construct were subject to this assay, we observed a strong correlation between the levels of both proteins among the best chemotactic cells (Fig. 7B,C), consistent with previous reports [17], [26] and with the proposed mechanism of noise reduction. We further observed that the levels of CheY-YFP and CheZ-CFP from the outer swarm ring, taken from populations with different average expression levels (Fig. 7B,C), collapse onto a single scatter plot (Fig. 7D). This result shows that not only the correlation between CheY and CheZ but also defined levels of these proteins around the native expression level (Fig. S6 in File S1) are important for optimal chemotaxis. Given linear scaling between kinase activity and CheY/CheZ concentration, our data suggest that the optimal adapted kinase activity is in the low range. As the native level of CheZ is similar to the level of CheA [28], substituting the measured rates 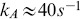 [29] and 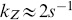 [31] in Eq. (14) suggests that in the adapted state only about 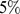 of receptor-kinase complexes are active. Higher adapted activity that was observed in previous experiments might have resulted from expression levels of the FRET pair above those of the wild-type proteins. The soft agar experiments also suggest that localization of CheZ to receptor clusters is not required for the proposed regulation of kinase activity by the levels of CheY/CheZ, since cells expressing CheZ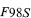-CFP that cannot bind CheA [25] show similarly strong selection for the correlated levels of CheY and CheZ (Fig. 7C). A higher optimal level of CheZ in these cells is consistent with lower CheZ activity in absence of localization to clusters.

Although we found strong evidence that chemosensing in *E. coli* follows noise compensation by exponential gain as presented in Eq. (4), it is not yet clear whether slowly varying multiplicative noise has any detrimental effect on chemotaxis efficiency. At this stage it cannot be decided whether the two different response behaviors shown in Fig. 2C result in different chemotaxis efficiency, as the areas under the response peaks are essentially equal. To address this question we focus on bacterial chemotaxis in shallow attractant gradients. In this regime changes in chemoeffector concentrations are small and cells can be expected to be strongly selected for noise suppression in order to increase the signal-to-noise ratio. For a stationary, linear attractant gradient, 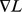, the steady state drift velocity in gradient direction, 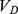, is given to leading order in 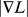 [31] by
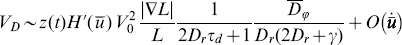
(19)with 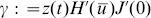 the effective adaptation rate, 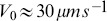 the swim velocity, 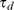 the time delay between ambient chemoeffector changes and flagella response, and 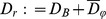, with 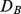 the Brownian rotational diffusion constant that changes swim orientation in agar by approximately 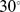 per second [15] (see File S1 for details). The overbar denotes averaging over many tumbling events and results in a time independent expression for the chemotaxis related diffusion constant, 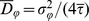, where 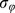 is the standard deviation of directional changes between successive swim runs of average duration 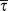. To compare the effect of different gain functions, 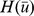, on the chemotactic efficiency in presence of multiplicative noise, 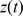, we define 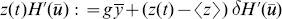, with 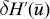 the deviation from the noise eliminating gain function 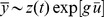 and 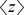 the expected value of 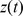. Here, the functional form of 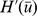 is chosen such that the absence of multiplicative noise, 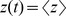, has the same effect as noise elimination. From Eq. (19) follows that for 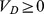 the drift velocity is a concave function in 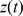, 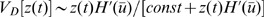, and therefore we get from Jensen’s inequality the relation 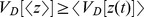, with equality only if 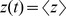. Consequently, the gain function 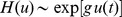 leads to highest chemotatic drift in the presence of slowly varying multiplicative noise and for environments where Eq. (19) applies.

## Discussion

The concept of noise compensation by exponential gain is designed to eliminate effects of slowly varying multiplicative noise on the output of a dynamical system that shows precise adaptation of the output signal. The concept requires a strict time scale separation between slow noise dynamics and fast relaxation of the output signal. In living systems, this concept can be realized by utilizing the natural time scale separation of cellular signaling: almost instantaneous conformation changes at the receptor level, fast signal transduction by phosphorylation of cytosolic proteins, and fast signal termination by feedback control in comparison to slow concentration changes of pathway components. It thereby provides a resource efficient alternative to noise reduction by increasing the abundance of signaling components and it is therefore not surprising that this concept has been likely realized in the highly sensitive chemotaxis pathway of *E. coli*. Recent experiments in this organism showed that chemoreceptor clusters assemble spontaneously and subsequently grow or shrink on time scales of minutes [18], [24]. As in *E. coli* a 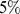 change in the number of functional receptor kinase complexes results in an almost 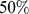 change in the adapted flagellar rotation bias due to the steep motor response curve [32], a tight control of the activated response regulator is essential for high chemotaxis efficiency.

The realization of noise compensation by exponential gain requires special pathway properties that have been observed in *E. coli* but could not be assigned a functional role. First, CheB phosphorylation as an indirect measure for the ratio of kinase and phosphatase activity is essential for this mechanism to work and would explain why CheB phosphorylation is found in almost all chemotactic prokaryotes [33], although CheB phosphorylation is non-essential for adaptation. Second, a competition between CheB and CheY for phosphate groups at CheA is required to feed back the information about any mismatch between the CheY/CheZ expression level and the adapted kinase activity. Third, fast phosphotransfer to CheY is required to ensure that the CheYp level is exclusively determined by the ratio between kinase activity and phosphatase acitivty (Eq. (14)), thus explaining the existence of a phosphoreceiver binding domain at CheA – a unique feature in bacterial two-component systems. Forth, the necessary linear relation between CheY (CheZ) expression level and kinase activity requires the concentration of phosphorylated CheB to scale as 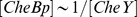, whereas CheY phosphorylation should not be affected by CheB abundance. This relation can give an explanation for the observed low copy number of CheB (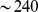 copies) in contrast to CheY (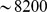 copies) [28], which makes CheY to the dominant phosphate sink. Precision in the adapted state further requires identical binding and co-localization dynamics of CheR and CheBp to reduce effects of fluctuations in receptor concentration and cluster size distribution [34]. It is therefore not surprising that CheR and CheBp have similar concentrations [28] and share the same docking domain at the Tsr and Tar receptors [35], with almost identical exchange dynamics between receptor clusters and cytosol as measured by FRAP [24].

Given the long evolutionary time span since radiation of the chemotaxis signaling system among prokaryotes, it can be expected that almost all evolutionary accessible mechanisms to increase the signal-to-noise ratio have been systematically scanned, and selected for their performance and resource efficiency. The outstanding signaling gain of order 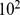 [16], [32] of the *E. coli* chemotaxis system is just one indication of this selection process, that most likely co-evolved with similar outstanding noise compensatory mechanisms, as presented in this work.

## Materials and Methods

### Strains and Plasmids

*Escherichia coli* K-12 strains VS100 [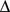 *cheY*], VS104 [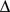
(*cheY cheZ*)], VS124 [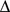
(*cheB cheY cheZ*)], VS127 [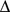 *cheR* 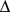
(*cheY cheZ*)] and VS149 [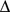
(*cheR cheB cheY cheZ*)] used in this work were described before [16], [36]. Strains LL4 [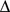 *flgM* 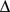
(*cheY cheZ*)] and LL5 [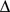 *flgM* 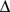
(*cheR cheB cheY cheZ*)] were constructed by the in-frame deletion of *flgM* in VS104 and VS149, respectively [6]. Strain VS162 that expresses CheY-YFP from the native chromosomal location was described before [6]. Plasmid pVS88 was used to co-express the CheY-YFP/CheZ-CFP FRET pair as one bicistronic mRNA under control of the inducible pTrc promoter [23]. pAV8 is a derivative of pVS88 encoding CheZ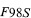-CFP (Vaknin & Berg, 2004). CheB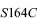-YFP was expressed under control of the pTrc promoter from pDK159 [38] and Tar-CFP (pDK53) [37], CheB (pVS91) (Liberman et al., 2004) and CheR (pVS113) (Lan et al., 2011) were expressed under control of the L-arabinose inducible pBAD promoter.

### Experimental Conditions

*E. coli* cells were grown under standard chemotaxis conditions (Sourjik & Berg, 2002) in a rotary shaker at 34°C to mid-exponential phase (OD600 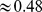) in tryptone broth (TB) medium supplemented with appropriate antibiotics. Expression was induced by indicated amounts of IPTG and arabinose. Swarm assays were performed at 34°C on TB plates supplemented with 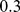
% agar (Applichem) and indicated concentrations of IPTG.

### FRET Measurements

Cell preparation, FRET measurements and evaluation of FRET data were performed as described previously, [16].

### Quantification of Gene Expression

Expression of fluorescent reporter proteins in individual cells was quantified as described before [6] using fluorescence imaging on a AxioImager fluorescence microscope equipped with an ORCA AG CCD camera (Hamamatsu) or by flow cytometry on a FACScan (BD Biosciences). Wild-type level of CheY was estimated based on strain VS162. Expression of untagged CheR and CheB was quantified using immunoblotting with a respective polyclonal antibody as described previously [31].

## Supporting Information

File S1 Derivation of mathematical expressions and issues of data analysis.
(PDF)
