# Supplementary material for: Exponential Signaling Gain at the Receptor Level Enhances Signal-to-Noise Ratio in Bacterial Chemotaxis
Source: PLoS One. 2014 Apr 15;9(4):e87815. doi: 10.1371/journal.pone.0087815 (PMC3988002; doi:10.1371/journal.pone.0087815)
Supplement: File S1 — Derivation of mathematical expressions and issues of data analysis. (PDF) [file pone.0087815.s001.pdf]

# Supplementary Information

## Contents


**1 Quantitative chemotaxis pathway model** **2**

**2 Chemotactic drift** **2**

2.1 Pathway response dynamics . . . . . 2

2.2 Drift in linear gradients . . . . . 5

2.3 Comparison with computer simulations . . . . . 7

**3 Molecular description of the chemotaxis pathway** **7**

3.1 Methylation dynamics . . . . . 8

3.2 Phosphorylation dynamics . . . . . 11

**4 Absence of CheZ dependence on kinase activity** **15**

**5 Cell-to-cell variability of receptor clusters** **16**

**6 Response behavior under exponential attractant ramps** **16**

**7 Data analysis** **17**

7.1 Quantification of gene expression . . . . . 17

7.2 Calibration of Imaging Scales . . . . . 19

# 1 Quantitative chemotaxis pathway model

The signal signaling gain of the *E. coli* chemotaxis pathway is modelled in this work by $g = 18$ receptor homodimers that form independent allosteric units and change activity in unison [1]. Here, the probability to find an active receptor complex takes the form $P(t) = [1 + \exp[-g\Delta\mathcal{F}]]^{-1}$, with $\Delta\mathcal{F} = -\epsilon + \epsilon'\langle m\rangle - f(L(t))$ ($\epsilon = 1.0, \epsilon' = 0.44$ – obtained from a linear fit to the data shown in Ref. [1]). $\Delta\mathcal{F}$ is a function of the average methylation level per receptor dimer, $\langle m\rangle$, and the free energy contribution of attractant binding, $f(L(t)) = \sum_i g_i/g[\ln(1+L(t)/K_i^{off}) - \ln(1+L(t)/K_i^{on})]$, with $L(t)$ a specific ligand concentration at time $t$ and $K_i^{on}$ and $K_i^{off}$ the corresponding dissociation constants of ligand binding to the $g_i$ receptors of type $i$ within an allosteric unit in the on and off state, respectively [1]. The global methylation dynamics follows the rate equation

$$\dot{\langle m\rangle} = F_1[P(t)] - F_2[P(t)]\frac{B_p(t)}{B^T} - F_3[P(t)]\frac{B^T - B_p(t)}{B^T} \tag{1}$$

with the activity dependent functions $F_i$, $i \in \{1,2,3\}$. Measurements of the methylation rate in the regime of low receptor activity are consistent with the approximation $F_1[P(t)] = const$ [2]. For demethylation, the situation is more complicated, as phosphorylation of CheB shows more than one order of magnitude higher demethylation activity but is non-essential for adaptation. Thus, $F_3$ is expected to show a monotone increasing dependency on $P(t)$, even in the low activity regime. However, the contribution $F_3$ can be neglected to good approximation for the native expression level of CheY [3] as the $\sim$ 70-fold more active phosphorylated form of CheB dominates the demethylation kinetics [4]. This is confirmed by a non-phosphorylatable mutant of CheB, expressed at the estimated native level, showing an order of magnitude higher adapted kinase activity than what is expected for wild type conditions (Fig. 4F and Fig. S3). Note that the high induction of the CheY-YFP/CheZ-CFP FRET pair (c.f. Fig. S5) leads to a significantly higher kinase activity, for which the $F_3$ contribution cannot be neglected any more. As the phosphoflux from CheA to CheB, $v_B$, determines the amount of phosphorylated CheB, $P(t) \sim v_B \sim B_p$, a linear dependency of the methylation rate on the mean receptor activity is the consequence, $\dot{\langle m\rangle} \sim const - P(t)$, if the $F_2$ term is approximately constant. This linear relation has been experimentally confirmed in the low activity regime [2].

# 2 Chemotactic drift

## 2.1 Pathway response dynamics

Bacterial chemotaxis results from a positive (negative) correlation between the average duration of a straight swimming run and the temporal change of attractant (repellent) concentration. The alternating periods of swimming (counter clockwise flagella rotation) and tumbling (clockwise flagella rotation) of a motile *E. coli* cell can be described to good approximation by a straight swimming cell that is subject to rotational diffusion. This is possible as the measured directional changes, $\varphi$, between swim runs are moderate,

with standard deviation $\sqrt{\langle\varphi^2\rangle} \approx 58^\circ$ [5]. We can therefore write the total rotational diffusion coefficient of the cell, $D_R = D_B + D_\varphi$, as a superposition of Brownian motion, $D_B \approx 0.068\,\mathrm{rad}^2\mathrm{s}^{-1}$ ($30^\circ$ directional change per second in agar, with viscosity 2.7 cp at $32^\circ C$ [6]), and stochastic tumbling events $D_\varphi = \langle\varphi^2\rangle/(4\tau(t))$ that are separated on average by the time span $\tau(t)$

$$D_R(t) = \underbrace{\frac{k_B T}{8\pi\eta a^3}}_{D_B} + \underbrace{\frac{\langle\varphi^2\rangle}{4\tau(t)}}_{D_\varphi} \quad . \tag{2}$$

Here, $k_B$ is the Boltzmann constant, $T$, the temperature, $\eta$ the solvent viscosity, and $a \approx 1\mu$m the effective hydrodynamics radius of an *E. coli* cell. For moderate chemoeffector gradients the average duration of a swim run, $\tau(t)$, shows only small changes for cells that have moved up or down the gradient [6]. This allows us to linearize around the average duration of a mean swim run, $\overline{\tau}$, in presence of a stationary chemoeffector gradient to give $\tau(t) = \overline{\tau} + \delta\tau$, with $\langle\delta\tau\rangle = 0$. The averaging, indicated by the brackets $\langle . \rangle$, runs over a sufficiently large amount of tumbling events and defines the quasi-stationary state as indicated by the overline. With the definitions $\overline{D}_\varphi = \langle\varphi^2\rangle/(4\overline{\tau})$ and $\overline{D}_R = D_B + \overline{D}_\varphi$ we get for Eq. (2) the expression

$$D_R(t) = \overline{D}_R - \overline{D}_\varphi \frac{\delta\tau(t)}{\overline{\tau}} \quad . \tag{3}$$

The temporal change in the probability to find a receptor in the active state, $P(t)$, depends on the encountered changes in chemoligand. In case of a stationary chemoeffector landscape this probability depends exclusively on the history of the swimming trajectory weighted by the loss of memory due to adaptation with rate $\gamma$ (see Methods section in the main text). From Eq. (9) of the main text we find after linearization around the stationary states $\overline{y} = z(t)H(\overline{u})$ and $f(\overline{L})$ the expression

$$\delta y(t) = -z(t)H'(\overline{u}) \int_{t_0}^{t} \delta\dot{f}(L(t'))\, e^{-z(t)H'(\overline{u})\epsilon'\lambda b\,(t-t')} dt' \tag{4}$$

$$\tag{5}$$

which results for the gain function $P(t) := H(u) = (1 + \exp[-N\,u])^{-1}$ in

$$\frac{\delta P(t)}{\overline{P}} = \frac{\delta y(t)}{\overline{y}} = -N[1-\overline{P}] \int_{t_0}^{t} \delta\dot{f}(L(t'))\, e^{-NP(t)[1-P(t)]\epsilon'\lambda b(t-t')} dt' \tag{6}$$

$$\approx -N \int_{t_0}^{t} \delta\dot{f}(L(t'))\, e^{-\gamma\,(t-t')} dt' \quad , \tag{7}$$

with , $\delta y = y(t) - \overline{y}$, $\delta f(L) = f(L) - f(\overline{L})$, and $\gamma := N\epsilon' r$. Here, $f(t) = \sum_i N_i/N[\ln(1 + L/K_i^{off}) - \ln(1 + L/K_i^{on})]$ is the per receptor free energy contribution of ligand binding that together with the free energy contribution from chemoreceptor methylation, $\mathcal{F}$, determines the activity state of the receptor cluster [1]. In Eq. (6) we have defined

the stationary receptor activity by $\overline{P} := P_a + \langle \delta P(t) \rangle$, where $P_a$ denotes the adapted receptor activity. We emphasize that the definition of a stationary receptor activity, $\overline{P}$, makes only sense in case of a sufficiently slowly varying extracellular chemoeffector concentration. The fraction of time the cell spends in clockwise rotation (CW) can be described to good approximation by the expression [7]

$$CW = \frac{{Y_p}^n}{{K_m}^n + {Y_p}^n} \tag{8}$$

with $n = 10$ the Hill coefficient of the motor response curve and $Y_p$ the concentration of phosphorylated CheY. With the definition of the clockwise bias $CW = \tau_R/(\tau + \tau_R)$ – with $\tau_R$ the average tumbling time – we arrive with a linear dependency between receptor activity, $P(t)$, and phosphorylated CheY, $Y_p(t) \approx y(t) = z(t)P(t)$, at the relation

$$\tau(t) = \tau_R \left( \frac{K_m}{Y_p(t)} \right)^n \approx \tau_R \left( \frac{K_m}{z(t)P(t-\tau_d)} \right)^n = \overline{\tau} \left( \frac{\overline{P}}{P(t-\tau_d)} \right)^n \quad . \tag{9}$$

Here, we have introduced with $\tau_d$ the response time needed for an extracellular change in chemoligand concentration to affect the swimming behavior of the cells. Note that $z(t-\tau_d) \approx z(t)$ by definition. For small fluctuation $\delta P(t)$ around the stationary activity state of the receptor clusters, $\overline{P}$, we find for the relative change in the mean swim duration the relation

$$\begin{aligned} \frac{\delta\tau(t)}{\overline{\tau}} &= -n \frac{\delta P(t-\tau_d)}{\overline{P}} \approx -n \int_{t_0}^{t} \tau_d^{-1} e^{-\tau_d^{-1}(t-t_1)} \frac{\delta P(t_1)}{\overline{P}} dt_1 & (10) \\ &= N\, n[1-\overline{P}] \int_{t_0}^{t} \tau_d^{-1} e^{-\tau_d^{-1}(t-t_1)} \int_{t_0}^{t_1} e^{-\gamma\,(t_1-t_2)}\, \delta\dot{f}(t_2)\, dt_2 dt_1 \quad . & (11) \end{aligned}$$

Here, we included the reasonable assumption that the signaling time delay $\tau_d$ is to good approximation the consequence of an exponentially decay of the phosphorylation and binding reactions. To calculate the drift velocity in a linear gradient we employ the approximation

$$\delta\dot{f}(t) \approx h[L(t^*)] \left( \dot{L}(t) - \langle \dot{L}(t) \rangle \right), \tag{12}$$

where the term $\langle \dot{L}(t) \rangle$ arises as $\delta\tau$ is measured relative to the stationary swim run duration $\overline{\tau}$. Here, $L(t^*)$ is the chemoattractant concentration at time $t^* \in [t_0, t]$ for a cell located in vicinity of the position $x(t^*)$ in direction of the gradient. The expansion coefficient, $h$, is given by

$$h[L(t^*)] = \left. \frac{\partial f}{\partial L} \right|_{L=L(t^*)} = \sum_i \frac{N_i}{N} \left[ \frac{1}{K_i^{off} + L(t^*)} - \frac{1}{K_i^{on} + L(t^*)} \right] \quad . \tag{13}$$

For stationary attractant gradients, $L(t^*) = \hat{L}(x(t^*))$, we can use the expression

$$\delta\dot{\hat{L}}(x(t^*)) = \nabla \hat{L}(x)|_{x=x(t^*)}\, (\dot{x}(t) - V_D) \tag{14}$$

with the attractant gradient at position $x$ given by $\nabla \hat{L}(x)$ and $V_D$ the drift velocity of the cell.

## 2.2 Drift in linear gradients

In the following we denote the current angle of the swim direction to the attractant gradient by $\theta(t)$. This allows us to rewrite the actual position of the cell, $x(t)$, in direction of an attractant gradient as

$$x(t) = V \int_{t_0}^{t} \cos\,\theta(t')dt' + x(t_0), \tag{15}$$

with $V$ the effective swim velocity along the trajectory. Thus, the expression for changes in the average swimming time can be rewritten with the help of Eq. (11) as

$$\frac{\delta\tau(t)}{\overline{\tau}} = N\,n[1-\overline{P}] \int_{t_0}^{t} \tau_d^{-1} e^{-\tau_d^{-1}(t-t_1)} \int_{t_0}^{t} e^{-\gamma\,(t_1-t_2)}\,\delta\dot{f}(t_2)\,dt_2 dt_1 \tag{16}$$

$$= \underbrace{N\,n[1-\overline{P}]h[L(t^*)]\nabla L}_{\alpha} \int_{t_0}^{t} \tau_d^{-1} e^{-\tau_d^{-1}(t-t_1)} \int_{t_0}^{t_1} e^{-\gamma\,(t_1-t_2)}\,[V\cos\theta(t_2) - V_D] dt_2 dt_1\ . \tag{17}$$

Further note that the quantity we are after – the net drift in gradient direction $V_D$ – is given by

$$V_D = \langle \dot{x}(t) \rangle = V\langle \cos(\theta) \rangle = V \int_\Omega \cos(\theta)\,P(\theta,t) d\Omega\ , \tag{18}$$

which requires the calculation of probability distribution, $P(\theta,t)$, to find the cell in orientation $\theta$ at time $t$. This probability distribution satisfies the rotational diffusion equation

$$\partial_t P(\theta,t) = -\mathbf{L}\cdot D_R(t)\mathbf{L}\,P(\theta,t) \tag{19}$$

with $\mathbf{L}$ the classical analog to the quantum mechanical angular momentum operator on the unit sphere $\mathbf{L} = -i\,\hat{\mathbf{r}} \times \nabla = i\left(\hat{\boldsymbol{\theta}}(\sin\,\theta)^{-1}\partial_\phi - \hat{\boldsymbol{\phi}}\partial_\theta\right)$. A formal solution to Eq. (19) is given by

$$P(\theta,t) = \mathcal{T}\exp\left[-\int_{t_0}^{t} \mathbf{L}\cdot D_R(t')\mathbf{L}\,dt'\right] P(\theta,t_0) \quad , \tag{20}$$

with $\mathcal{T}$ the time ordering operator. In the following we expand the solution of $P(\theta,t)$ to first order in the smallness parameter $\overline{D}_\varphi\,\delta\tau(t)/\overline{\tau}$ by employing Eq. (3) and using Dyson series

$$\mathcal{T}\exp\left[-\int_{t_0}^{t} \mathbf{L}\cdot D_R(t')\mathbf{L}dt'\right] = \hat{U}(t,t_0) + \int_{t_0}^{t} \hat{U}(t,t')\mathbf{L}\cdot\overline{D}_\varphi\,\frac{\delta\tau(t')}{\overline{\tau}}\mathbf{L}U(t',t_0)dt' + \mathcal{O}\left(\frac{\delta\tau(t')}{\overline{\tau}}\right)^2$$

with

$$\hat{U}(t,t_0) := \exp\left[-\int_{t_0}^{t} \mathbf{L}\cdot\overline{D}_R\mathbf{L}\,dt'\right]. \tag{21}$$

This expansion allows us to give an accurate, explicit expression for the chemotaxis drift velocity, $V_D$, for moderate gradients

$$
\begin{aligned}
\frac{V_D}{V} &= \int_\Omega \cos(\theta) P(\theta,t) d\Omega && (22)\\
&= \mathcal{T} \int_\Omega \cos(\theta) \exp\left[-\mathbf{L}\cdot\int_{t_0}^{t} D_R(t')\mathbf{L}dt'\right]\frac{d\Omega}{4\pi} && (23)\\
&\approx \underbrace{\int_\Omega \cos(\theta)\hat{U}(t,t_0)\frac{d\Omega}{4\pi}}_{=0} + \mathcal{T}\int_\Omega\int_{t_0}^{t}\cos(\theta)\hat{U}(t,t_1)\,\mathbf{L}\cdot\overline{D}_\varphi\frac{\delta\tau(t_1)}{\overline{\tau}}\mathbf{L}\,\hat{U}(t_1,t_0)dt_1\frac{d\Omega}{4\pi} && (24)\\
&= \int_\Omega\int_{t_0}^{t}\cos(\theta)\hat{U}(t,t_1)\,\overline{D}_\varphi\mathbf{L}^2\frac{\delta\tau(t_1)}{\overline{\tau}}\,\hat{U}(t_1,t_0)dt_1\frac{d\Omega}{4\pi} && (25)\\
&= 2\overline{D}_\varphi\alpha\int_\Omega\int_{t_0}^{t}\cos(\theta)\hat{U}(t,t_1)\int_{t_0}^{t_1}\hat{U}(t_1,t_2)\,\tau_d^{-1}e^{-\tau_d^{-1}(t_1-t_2)} && (26)\\
&\qquad *\int_{t_0}^{t_2}\hat{U}(t_2,t_3)e^{-\gamma\,(t_2-t_3)}\,[V\cos\theta(t_3)-V_D]\hat{U}(t_3,t_0)dt_3dt_2\,dt_1\frac{d\Omega}{4\pi} && (27)\\
&= \frac{2\overline{D}_\varphi\alpha}{3}\int_{t_0}^{t}e^{-2\overline{D}_R(t-t_1)}\,\tau_d^{-1}\int_{t_0}^{t_1}e^{-(2\overline{D}_R+\tau_d^{-1})\,(t_1-t_2)}\int_{t_0}^{t_2}e^{-(2\overline{D}_R+\gamma)\,(t_2-t_3)}\,V\,dt_3dt_2dt_1\\
&\overset{t\gg\overline{\tau}}{=} \frac{V\alpha}{3}\frac{\overline{D}_\varphi}{\overline{D}_R}\frac{\tau_d^{-1}}{2\overline{D}_R+\tau_d^{-1}}\,\frac{1}{2\overline{D}_R+\gamma} && (28)\\
&= \frac{V\alpha}{3}\frac{\overline{D}_\varphi}{D_B+\overline{D}_\varphi}\frac{1}{2(D_B+\overline{D}_\varphi)\tau_d+1}\,\frac{1}{2(D_B+\overline{D}_\varphi)+\gamma}\quad. && (29)
\end{aligned}
$$

Due to interruption by tumbling events, a reduced effective swim velocity, $V$, is the consequence, which is given to leading order by $V = \frac{\overline{\tau}}{\overline{\tau}+\tau_R}V_0$, with $V_0$ the swimming velocity of the cell. We thus arrive at the final expression for the drift velocity,

$$
\frac{V_D}{V} = \frac{V_0\alpha}{3}\frac{\overline{\tau}}{\overline{\tau}+\tau_R}\,\frac{1}{2(D_B+\overline{D}_\varphi)\tau_d+1}\,\frac{\overline{D}_\varphi}{D_B+\overline{D}_\varphi}\,\frac{1}{2(D_B+\overline{D}_\varphi)+\gamma} \qquad (30)
$$

with $\tau_R = 0.14\,\mathrm{s}$ the average tumbling time. Our analysis shows that the time a cells spends in the tumbling mode should be minimized to arrive at highest chemotactic drift. However, minimizing tumbling times means that eventually rotational Brownian motion will dominate directional changes, overriding the chemotaxis strategy to prolong swimming when moving towards favorable environments and to shorten swim run duration when moving away from favorable environments. Therefore, swim run duration should dominate the tumbling duration but should remain far below the average time needed for a cell to reverse direction due to Brownian motion, $\tau_B$. As the adaptation time should be long enough to allow for reliable comparison of chemoeffector concentrations at different times but short enough to cut off any information that is not correlated to the actual swimming we arrive at the relation $\tau_R < \overline{\tau}, \gamma^{-1} < \tau_B$ for highly chemotactic cells.

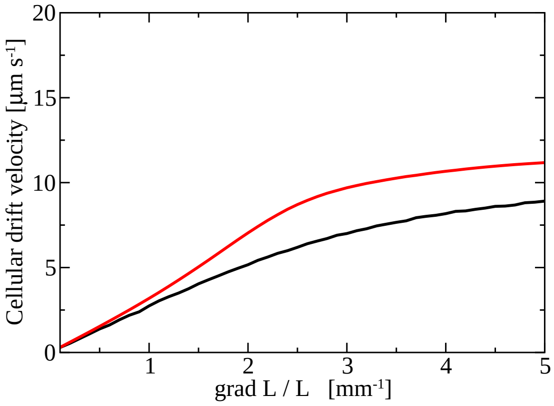


Figure S1: Chemotactic drift velocity in dependence on the gradient strength. The stimulated cells start drifting at an ambient concentration of 10$\mu$M methylaspartate. The analytic results (red line) are shown in comparison to computer simulations (black line).

### 2.3 Comparison with computer simulations

To show the regimes of validity of the analytical approximation for the net drift velocity in direction of an attractant gradient we employed computer simulations to generate swimming trajectories of individual cells. To this end we integrated Eq. (4) numerically using equidistant time steps of 0.005 seconds. After every time step the probability to enter or leave a tumbling and swimming mode is calculated. The mean duration of swim run in the adapted state is fixed to 0.8072s and the adaptation rate is set to 0.4022s$^{-1}$. The later corresponds to an effective methyltransferase activity of $k_R R = 0.05674\text{s}^{-1}$. In the computer simulations, swim and tumbling duration are taken from exponential distributions, to generate similar statistics as experimentally observed [8, 9]. The tumbling angle between swim runs is chosen to be Gaussian distributed with a standard deviation of 58° and rotational diffusion changes swimming direction by 30° per second. To allow an easy comparison we set the signaling time to zero, $\tau_d = 0$.

## 3 Molecular description of the chemotaxis pathway

The central argument why mass action equations are sufficient to describe cellular signal pathways (see below) is a consequence of intracellular noise reduction. As the effect of noise is always linked to both molecule copy number and the reaction time scale, we expect that the fast response times of the pathway, $\tau \sim 10^{-1}$s, requires sufficiently high abundance of the proteins per cell (strain RP437, [3]) of CheY ($\sim$ 8200 copies), CheZ ($\sim$ 3200 copies), and CheA (long isoform) ($\sim$ 4500 copies). In contrast the more than one order of magnitude slower adaptation kinetics can buffer the small concentration

for CheB ($\sim$ 240 copies) and CheR ($\sim$ 140 copies) [3]. We emphasize that noise due to low copy number of the protein CheR is measurable but influences only to small extend the chemotactic efficiency of a population [10]. To determine the protein concentration we used an average cytosolic cell volume of 0.9fl. This amounts in the concentrations: [CheY]$\approx$ 15.1$\mu$M, [CheZ]$\approx$ 5.9$\mu$M, [CheA]$\approx$ 8.3$\mu$M, [CheB]$\approx$ 0.44$\mu$M, and [CheR]$\approx$ 0.26$\mu$M.

## 3.1 Methylation dynamics

The molecular mechanisms of how the average cellular receptor activity, $P(t)$, drives the methylation rates are currently not fully understood. However the leading order functional dependencies of the adaptation dynamics can be determined from response measurements of this work. In the following we make use of the fact that the phosphorylation dynamics is much faster than the methylation dynamics and thus can be treated as equilibrated on the adaptation time scale. The mass action equations for the methylation dynamics of receptors of type $\alpha \in \{1, 2, 3, 4\}$, (e.g. Tar, Tap, Tsr, Trg) in the methylation state $m$, are given by

$$\begin{aligned} \partial_t T_m^{(\alpha)}(t) &= k_R\, \mathcal{H}_1\left[R^T\right] \mathcal{H}_2\left[P(t)\right] \left(T_{m-1}^{(\alpha)}(t) - T_m^{(\alpha)}(t)\right) && (31) \\ &\quad + k_B\, \mathcal{G}_1\left[B^T\right] \mathcal{G}_2\left[P(t)\right] \left(T_{m+1}^{(\alpha)}(t) - T_m^{(\alpha)}(t)\right) && (32) \end{aligned}$$

with $\mathcal{H}_1$ and $\mathcal{G}_1$ monotone increasing functions of the total concentrations of the methyltransferase CheR, $R^T$, and methylesterase CheB, $B^T$, respectively, and $\mathcal{H}_2$ and $\mathcal{G}_2$ the unknown dependencies on the probability to find a receptors in the active state, $P(t)$, at time $t$. Averaging Eq. (31) over all receptor types and methylation states results in

$$\partial_t \langle m\rangle(t) = k_R\, \mathcal{H}_1\left[R^T\right] \mathcal{H}_2\left[P(t)\right] - k_B\, \mathcal{G}_1\left[B^T\right] \mathcal{G}_2\left[P(t)\right] \quad (33)$$

with $\langle m\rangle = \sum_{\alpha,m} m\, T_m^{(\alpha)} / \sum_{\alpha,m} T_m^{(\alpha)}$, assuming that the highest methylation state is not significantly populated within the physiological regime. Note that there must not exist any dependency on the methylation state in Eq. (33) as a consequence of perfect adaptation [11]. The functional form of $\mathcal{H}_i$ and $\mathcal{G}_i$, $i \in \{1, 2\}$ can be inferred from experiments, shown in Fig. 2 in the main text. Here, an increase in CheR abundance leads to both an increase in the activity [12] and the adaptation rate, whereas an increase in CheB reduces kinase activity but leaves the adaptation rate unchanged. As the probability to find a receptor in the active state, $P(t)$, equilibrates rapidly and is determined by the chemoattractant concentration, $L$, and the average methylation level, $\langle m\rangle$, the receptor activity has on the mean field level a functional time dependence given by $P(t) = P\left(\langle m\rangle(t), L(t)\right)$. By taking the time derivative, $\partial_t P(t) = \partial_{\langle m\rangle} P\ \partial_t \langle m\rangle(t) + \partial_L P\ \partial_t L(t)$, we can rewrite Eq. (33) as

$$\partial_t P(t) = \partial_{\langle m\rangle} P(t) \left\{ k_R\, \mathcal{H}_1\left[R^T\right] \mathcal{H}_2\left[P(t)\right] - k_B\, \mathcal{G}_1\left[B^T\right] \mathcal{G}_2\left[P(t)\right] \right\} + \partial_L P(t)\ \partial_t L(t). \quad (34)$$

From Fig. 2A in the main text follows that multiplication of Eq. (34) by the total concentration of CheB, $B^T$, shows identical adaptation dynamics over one oder of magnitude in CheB abundance. Therefore a transformation of Eq. (34) to a scaled activity, $P_B(t) := P(t)B^T$, must not show any explicit dependency on $B^T$ to leading order,

$$\begin{aligned}\partial_t P_B(t) &= \partial_{\langle m\rangle} P_B(t)\left\{k_R\,\mathcal{H}_1\left[R^T\right]\mathcal{H}_2\left[P_B(t)/B^T\right] - k_B\,\mathcal{G}_1\left[B^T\right]\mathcal{G}_2\left[P_B(t)/B^T\right]\right\}\\ &\quad +\partial_L P_B\;\partial_t L(t)\quad . \end{aligned} \tag{35}$$

Independence of the solution $P_B(t)$ on the CheB concentration requires the functional forms $\mathcal{H}_2 = 1$ and $\mathcal{G}_1[x] \propto \mathcal{G}_2[x] = x^\alpha$, with a yet unknown exponent $\alpha$, which results in

$$\partial_t P_B(t) = \partial_{\langle m\rangle} P_B(t)\left\{k_R\mathcal{H}_1\left[R^T\right] - k_B\,b\,(P_B(t))^\alpha\right\} + \partial_L P_B(t)\;\partial_t L(t)\quad , \tag{36}$$

where we have introduced the proportionality constant $b$. For the adapted state ($\partial_t P_B(t) = 0$ and $\partial_t L(t) = 0$) follows that $k_R\mathcal{H}_1\left[R^T\right] - k_B\,b\,(P_B^a)^\alpha = 0$ or $(P^a)^\alpha = k_R\mathcal{H}_1\left[R^T\right]/(k_B\,b\,\left(B^T\right)^\alpha)$. As the adapted state is unchanged for concerted overexpression of CheR and CheB (data not shown) we obtain the equation

$$(P^a)^\alpha = \frac{k_R\,r\,\left(R^T\right)^\alpha}{k_B\,b\,\left(B^T\right)^\alpha}\ , \tag{37}$$

where we introduced the proportionality constant $r$. The yet unspecified derivatives of $P_B(t)$ with respect to $\langle m\rangle$ and $L$ must be functions that depend uniquely on $P_B(t)$, $\partial_{\langle m\rangle} P_B(t) = f_m[P_B(t)]$ and $\partial_L P_B(t) = f_L[P_B(t)]$, because of the dynamic scaling property Fig. 2B in the main text. We therefore arrive at the equation

$$\partial_t P_B(t) = f_m[P_B(t)]\left\{k_R\,r\left(R^T\right)^\alpha - k_B\,b\,(P_B(t))^\alpha\right\} + f_L[P_B(t)]\;\partial_t L(t) \tag{38}$$

As the adaptation rate increases linear with $R^T$ [12] (see also Fig. 2E in the main text) and $P_B^a \propto R^T$ (Fig. S2) to good approximation in the low activity regime, we observe to leading order invariance of the function $\hat{P}_B(\tau) := P_B(\tau/R^T)/R^T$, with $\tau := t\,R^T$, for different concentrations of CheR and stepwise changes in attractant concentration. Multiplication of Eq. (38) by $(R^T)^{-2}$ gives

$$\begin{aligned}\partial_\tau \hat{P}_B(\tau) &= (R^T)^{-2} f_m[P_B(\tau/R^T)]\left\{k_R\,r\left(R^T\right)^\alpha - k_B\,b\left(P_B(\tau/R^T)\right)^\alpha\right\}\\ &\quad +(R^T)^{-1} f_L[P_B(\tau/R^T)]\;\partial_\tau L(\tau/R^T)\quad . \end{aligned} \tag{39}$$

Note that a step change of attractant with magnitude $\Delta L$ at time $t = 0$ results in the expression $\partial_t L(t) = \Delta L\,\delta(t) = \Delta L\,\delta(\tau/R^T) = R^T\Delta L\,\delta(\tau)$, with $\delta(t)$ the delta distribution. Elimination of all dependencies on CheR from Eq. (39) requires the remaining unknown functionals to take the forms $f_m(x) \propto x^{2-\alpha}$ and $f_L(x) = l\,x$. A least squares fit (see Fig. 2 in the main text) reveals that the choice $\alpha = 1$ reproduces accurately the response of *E. coli* to chemoeffectors

$$\partial_\tau \hat{P}_B(\tau) = \hat{P}_B(\tau)\left\{k_R\,r - k_B\,b\hat{P}_B(\tau)\right\} + l\,\hat{P}_B(\tau)\;\Delta L\,\delta(\tau)\ , \tag{40}$$

where we have to introduce a negative proportionality constant, $l < 0$, if $L(t)$ reflects the ambient attractant concentration. Resubstitution gives the final result

$$\partial_t P_B(\tau) \;=\; P_B(t)\left\{k_R\, r R^T - k_B\, b P_B(t)\right\} + l\, P_B(t)\;\Delta L\, \delta(t)\ . \tag{41}$$

This logistic differential equation reproduces accurately the response dynamic as shown in Fig. 2 in the main text by the solid lines. The solution of Eq. (41) reads

$$P(t) \;=\; \frac{k_R\, r R^T}{k_B\, b B^T}\left[1 + \left(e^{-l\,\Delta L} - 1\right) e^{-k_R\, r R^T\, t}\right]^{-1} \quad \forall\; t > 0 \tag{42}$$

This result shows that the time needed to recover the pre-stimulus value depends on the methyltransferase concentration, $R^T$, but not on the methylesterase concentration, $B^T$. So far we have given very strong evidence that the integral feedback that leads to adaptation is mediated by the activity dependent term $k_B\, b\, B^T P(t)$. For this term there exists experimental evidence that CheB phosphorylation is the dominant but not the only contribution. This is because *E. coli* cells show adaptation to chemoeffectors for a CheB mutant that cannot be phosphorylated. We conjecture that there is second contribution resulting an integral feedback where CheB bind with weak affinity to active receptors. Thus there is evidence that the integral feedback derived from the scaling arguments above is biochemically realized by two mechanisms, resulting in $k_B B^T P(t) = k'_B Bp + k''_B(B^T - Bp)P(t)$, with demethylation rates $k'_B \gg k''_B$ (see Methods section in the main text)

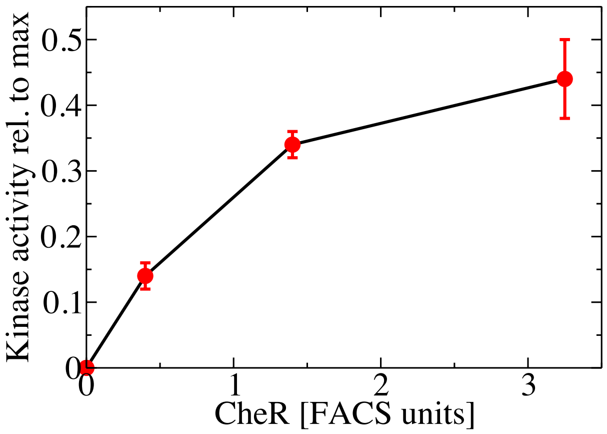


Figure S2: Increase of kinase activity with CheR under control of a BAD promoter inducible by L-arabinose. The expression level of CheR for different concentrations of L-arabinose is determined from FACS measurements, using a CheR-YFP fusion construct.

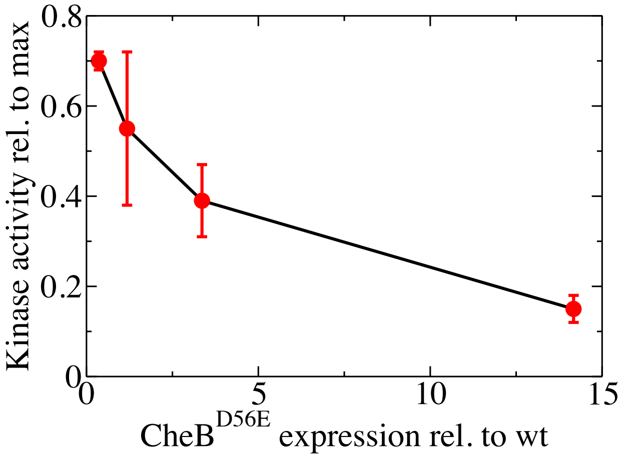


Figure S3: Decrease of kinase activity with expression level of a non-phosphorylatable CheB mutant CheB$^{D56E}$ under control of a BAD promoter inducible by L-arabinose. The CheB$^{D56E}$ expression levels relative to the native CheB expression level were determined by Western Blotting.

## 3.2 Phosphorylation dynamics

The phosphorylation dynamics of CheY has be extensively studied in *E. coli.* Phosphorylation takes place at the kinase CheA, which consists of a $P_2$ binding domain and a $P_1$ domain that transfers phospho-groups to CheY and CheB. Both CheY and CheB are most effectively phosphorylated by binding to the $P_2$ domain but can also receive phospho-groups by a direct phosphotransfer from the $P_1$ domain. Transfer of phospho-groups from the ATP binding pocket to the $P_1$ domain is believed to be the rate limiting step in the phosphorylation cascade and dephosphorylation by CheZ is the by far strongest contribution to CheYp hydrolysis. This gives rise to the equation

$$k_A A P(t) \approx k_Z Z^T \frac{Y_p}{K_Z + Y_p} \tag{43}$$

with $Z^T$ the total concentration of CheZ phosphatases, $k_Z$ the dephosphorylation rate, $K_Z$ the corresponding Michaelis-Menten constant, $A$ the concentration of non occupied kinases associated to fully functional receptor complexes and $P(t)$ denotes the average receptor activity. The whole phosphorylation network is presented in the following.

We assume that autophosphorylation of the kinase CheA depends on the average receptor activity $P(t)$, and thus gives rise to the reaction equation

$$A + ATP \xrightarrow{k_A P(t)} Ap + ADP \quad . \tag{44}$$

Phosphorylation of the response regulators CheY and CheB predominantly takes place by binding to the $P_2$ domain of CheA and subsequent phosphotransfer from the phospho-

rylated $P_1$ domain to CheY and CheB, respectively. This is summarized in the reaction equations

$$Y + Ap \underset{k_Y^-}{\overset{k_Y^+}{\rightleftharpoons}} [YAp] \xrightarrow{k_Y} Yp + A \tag{45}$$

$$B + Ap \underset{k_B^-}{\overset{k_B^+}{\rightleftharpoons}} [BAp] \xrightarrow{k_B} Bp + A\ . \tag{46}$$

Finally, dephosphorylation of CheYp is mediated by the phosphatase CheZ

$$Yp + Z \underset{k_Z^-}{\overset{k_Z^+}{\rightleftharpoons}} [YpZ] \xrightarrow{k_Z} Y + Z + p_i\ , \tag{47}$$

whereas phosphorylated CheB autodephosphorylates

$$Bp \xrightarrow{\mu_B} B + p_i. \tag{48}$$

Applying the law of mass action, we obtain the set of rate equations

$$\frac{d}{dt}Ap = k_A P(t) A - k_Y^+ Y Ap + k_Y^- [YAp] - k_B^+ B Ap + k_B^- [BAp] \tag{49}$$

$$\frac{d}{dt}Yp = k_Y [YAp] - k_Z^+ Yp Z + k_Z^- [YpZ] \tag{50}$$

$$\frac{d}{dt}[YAp] = k_Y^+ Y Ap - (k_Y^- + k_Y)[YAp] \tag{51}$$

$$\frac{d}{dt}[YpZ] = k_Z^+ Yp Z - (k_Z^- + k_Z)[YpZ] \tag{52}$$

$$\frac{d}{dt}Bp = k_B [BAp] - \mu_B Bp \tag{53}$$

$$\frac{d}{dt}[BAp] = k_B^+ B Ap - (k_B^- + k_B)[BAp], \tag{54}$$

where complexes are denoted by brackets. These equations are completed by the conservation laws

$$A^c = A + Ap + [YAp] + [BAp] \tag{55}$$

$$Y^T = Y + Yp + [YAp] + [YpZ] \tag{56}$$

$$Z^T = Z + [YpZ] \tag{57}$$

$$B^T = B + Bp + [BAp]. \tag{58}$$

Here, $A^c$ denotes the total concentration of kinases associated to fully functional receptor complexes. Due to the small amount of CheB, i.e. $B^T \ll A^c$ and $B^T \ll Y^T$, we can neglect all interactions of CheB with CheA in the differential equation governing the dynamics of the phosphorylation state of CheA. Hence, instead of Eqs. (49) and (55) we consider

$$\frac{d}{dt}Ap \approx k_A P(t) A - k_Y^+ Y Ap + k_Y^- [YAp] \tag{59}$$

and

$$A^c \approx A + Ap + [YAp]. \tag{60}$$

If we assume a quasi-steady state approximation for the formation of complexes, i.e. $\frac{d}{dt}[YAp] \approx 0$, $\frac{d}{dt}[BAp] \approx 0$, $\frac{d}{dt}[YpZ] \approx 0$ and introduce the new dynamic variables

$$\tilde{Y}p = Yp + [YpZ] \tag{61}$$

$$\tilde{A}p = Ap + [YAp] \tag{62}$$

we get the set of differential algebraic equations

$$\frac{d}{dt}\tilde{A}p = k_A P(t)(A^c - \tilde{A}p) - k_Y[YAp] \tag{63}$$

$$\frac{d}{dt}\tilde{Y}p = k_Y[YAp] - k_Z[YpZ] \tag{64}$$

$$\left(Y^T - \tilde{Y}p - [YAp]\right)\left(\tilde{A}p - [YAp]\right) - K_Y[YAp] = 0 \tag{65}$$

$$\left(\tilde{Y}p - [YpZ]\right)\left(Z^T - [YpZ]\right) - K_Z[YpZ] = 0 \tag{66}$$

$$\frac{d}{dt}Bp = k_B[BAp] - \mu_B Bp \tag{67}$$

$$\left(B^T - Bp - [BAp]\right)\left(\tilde{A}p - [YAp]\right) - K_B[BAp] = 0, \tag{68}$$

where we used the set of conservation laws (56)-(58) and (60) and introduced the Michaelis-Menten constants

$$K_i = \frac{k_i^- + k_i}{k_i^+}, \qquad i = Y, B, Z \tag{69}$$

Phosphorylation dynamics is much faster than methylation of the receptor. Thus we can assume that phosphorylation is equilibrated on the timescale the average receptor activity $P(t)$ changes due to methylation. To obtain this quasi steady state, we have to solve the set of algebraic equations

$$k_A P(t)(A^c - \tilde{A}p) - k_Y[YAp] = 0 \tag{70}$$

$$k_Y[YAp] - k_Z[YpZ] = 0 \tag{71}$$

$$\left(Y^T - \tilde{Y}p - [YAp]\right)\left(\tilde{A}p - [YAp]\right) - K_Y[YAp] = 0 \tag{72}$$

$$\left(\tilde{Y}p - [YpZ]\right)\left(Z^T - [YpZ]\right) - K_Z[YpZ] = 0 \tag{73}$$

$$k_B[BAp] - \mu_B Bp = 0 \tag{74}$$

$$\left(B^T - Bp - [BAp]\right)\left(\tilde{A}p - [YAp]\right) - K_B[BAp] = 0. \tag{75}$$

Since Eqs. (70)-(73) are independent of Eqs. (74) and (75), we can solve them first. If we assume $A^c - \tilde{A}p \approx A^c - [YAp]$, i.e. neglecting the contribution of $Ap$ in Eq. (70), we obtain for $Ap$ to leading order in $P(t)$

$$Ap = \frac{k_A K_Y A^c}{k_Y Y^T} P(t) + \mathcal{O}(P(t)^2). \tag{76}$$

Since $A_p = \tilde{A}p - [YAp]$, we plug this into Eq. (75) and solve Eqs. (74) and (75) for $Bp$ and $[BAp]$, respectively. This results in

$$Bp \approx \frac{k_A k_B K_Y A^c B^T P(t)}{k_A(k_B + \mu_B) K_Y A^c P(t) + k_Y \mu_B K_B Y^T} = \frac{\frac{k_A}{\lambda_B}\frac{A^c}{Y^T} B^T P(t)}{\chi \frac{k_A}{\lambda_B}\frac{A^c}{Y^T} P(t) + 1} \tag{77}$$

where we introduced

$$\Lambda = \frac{k_Y K_B}{k_B K_Y} \tag{78}$$

$$\lambda_B = \mu_B \Lambda \tag{79}$$

$$\chi = \frac{k_B + \mu_B}{k_B}. \tag{80}$$

The phosphotransfer rate from CheA to CheB to leading order in $P(t)$ is given by

$$k_B[BAp] \approx \frac{k_A}{\Lambda}\frac{A^c}{Y^T} B^T P(t) + \mathcal{O}(P(t)^2) \tag{81}$$

In the adapted state, i.e. $\dot{P}(t) = 0$, it follows from Eq. (6) in the main text that

$$k_R R^T = k_B Bp \tag{82}$$

holds and thus, using Eq. (77), we get

$$\frac{k_R}{k_B} R^T \approx \frac{\frac{k_A}{\lambda_B}\frac{A^c}{Y^T} B^T P^a}{\chi \frac{k_A}{\lambda_B}\frac{A^c}{Y^T} P^a + 1}, \tag{83}$$

with $P^a$ the adapted receptor activity. Solving for $P^a$ yields

$$P^a \approx \frac{\frac{k_R}{k_B} R^T Y^T}{\frac{k_A}{\lambda_B} A^c B^T \left(1 - \chi \frac{k_R}{k_B}\frac{R^T}{B^T}\right)} \approx \frac{k_R \lambda_B}{k_B k_A}\frac{R^T Y^T}{B^T A^c}. \tag{84}$$

From Eqs. (70) and (71)

$$k_A P(t)(A^c - \tilde{A}p) = k_Z[YpZ] \tag{85}$$

follows. Making the same assumption as above, i.e. $A^c - \tilde{A}p \approx A^c - [YAp]$ we get

$$k_A P(t)(A^c - [YAp]) = k_A A^c \frac{P(t)}{1 + \frac{k_A}{k_Y} P(t)}. \tag{86}$$

If we assume that $k_A \ll k_Y$ and since by definition $P(t) \leq 1$ holds, we get

$$k_A P(t)(A^c - [YAp]) \approx k_A P(t) A^c. \tag{87}$$

The complex $[YpZ]$ can be written as a function of $Yp$ and $Z^T$,

$$[YpZ] = Z^T \frac{Yp}{K_Z + Yp}. \tag{88}$$

Using Eq. (87) and (88) we get for (85)

$$k_A P(t) A^c \approx Z^T \frac{Yp}{K_Z + Yp}. \tag{89}$$

We obtain for the adapted state by using the approximation for $P^a$ derived in (84)

$$\frac{k_R \lambda_B}{k_B k_Z} \frac{R^T Y^T}{B^T Z^T} \approx \frac{Yp}{K_Z + Yp} \tag{90}$$

independent of $k_A$ and $A^c$.

## 4 Absence of CheZ dependence on kinase activity

To show that CheZ does not alter kinase activity we employed the fact that phosphorylated CheB strongly localizes at the receptor complexes [13]. By substituting native CheB by a catalytic inactive mutant that is fluorescently tagged, CheBS164C-YFP, we were able to measure CheBS164C-YFP localization at the receptors by employing the aspartate receptor fusion construct Tar-CFP. Localization is proportional to the FRET signal Tar-CFP/CheBS164C-YFP and can be compared, after stimulation with methylaspartate, with the localization of unphosphorylated CheBS164C-YFP. We employed a CheY deleted strains to avoid any interference between the two phospho-receivers CheB and CheY. The FRET amplitude arising from the difference between the FRET signal before and after the stimulus shows no significant difference if CheZ is present at native level or absent: $0.0154 \pm 0.0027$ for the $\Delta$CheY strain and $0.0162 \pm 0.0032$ for the $\Delta$CheYZ strain. Kinase activities for three independent experiments for each strain are listed in the table below.

| Exp. No. | kinase activity (AU), $\Delta$CheY | kinase activity (AU), $\Delta$CheYZ |
|---|---|---|
| 1 | 0.0163 | 0.0151 |
| 2 | 0.0175 | 0.0136 |
| 3 | 0.0124 | 0.0198 |

Table S1

## 5 Cell-to-cell variability of receptor clusters

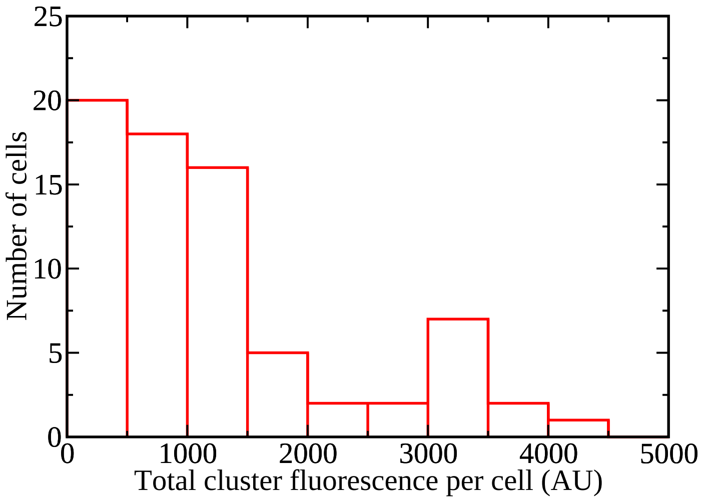


Figure S4: Histogram of the cell-to-cell variability of the total amount of receptors that are organized in clusters. The variability is significantly higher than expected from gene expression noise alone, as additional noise contributions arise from stochasticity in receptor cluster assembly and uneven distribution of cluster among daughter cells.

## 6 Response behavior under exponential attractant ramps

To test the quality of the mathematical model shown in the Box of the main text we reproduced the response behavior of the flagellar rotation bias upon temporal changes in attractant ($\alpha$-methyl-DL-aspartate). The flagella rotation bias is a highly significant measure of the kinase activity and has the advantage not to rely on fluorescent fusion constructs. Fig. 1 shows the change in counter clockwise (CCW) bias in exponentially increasing (upper red area) or exponentially decreasing (lower red area) attractant concentrations over time. For the calculation we employed the linearized version of the free energy contribution of ligand binding $\dot{\mathcal{S}}(t) \approx h(\hat{L})\dot{L}(t)$. The red areas arise from inserting the whole range of possible expansion parameters $\hat{L} \in [43.2\mu M, 448\mu M]$. Here, $43.2\mu$M is the initial attractant concentration and $448\mu$M is the final attractant concentrations. The attractant concentration at time $t$ is given by $L(t) \propto \exp[a\,t]$, with $a$ the ramp rate [8, 9]. We employed an adaption rate $\gamma = 0.4\,s^{-1}$ for 36°C that has been inferred from FRET measurements of the kinase activity. Note that the adaptation rate is highly temperature dependent and decreases approximately eight fold at 20°C,

$\gamma = 0.05\, s^{-1}$.

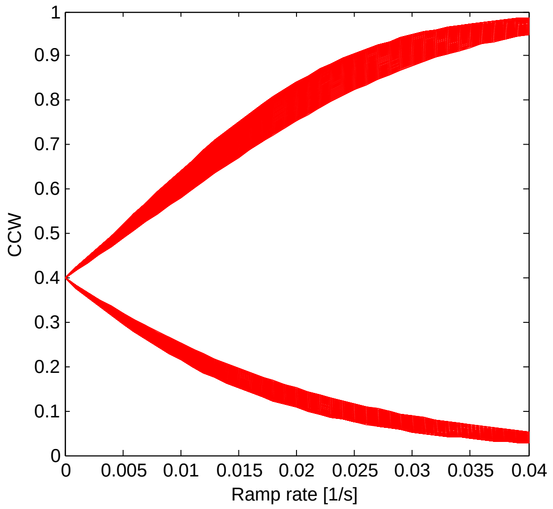


Figure S5: CCW bias of flagellar rotation in dependence on the rate of an exponentially changing attractant concentration [8].

# 7 Data analysis

## 7.1 Quantification of gene expression

The gene fusion constructs *cheY-yfp* and *cheZ-cfp* were expressed as a bicistronic mRNA from a single plasmid (pVS88) under the control of an IPTG-inducible promoter pTrc. The dependence of the expression levels on the IPTG concentration can be fitted by a Hill function with an offset,

$$\text{YFP\_facs} = p_1 \frac{\text{IPTG}^{p_4}}{\text{IPTG}^{p_4} + p_2^{p_4}} + p_3, \quad p_1 = 245 \pm 31, p_2 = 35 \pm 8, p_3 = 6 \pm 10, p_4 = 1.5 \pm 0.4. \tag{91}$$

where the offset originates from the leaky expression of the pTrc promoter.

| IPTG [$\mu$M] | CheY-YFP (FACS) | CheY-YFP [$\mu$M] |
|---|---|---|
| 0 | $6 \pm 10$ | $3.3 \pm 5.5$ |
| 10 | $40 \pm 9$ | $22.0 \pm 8.0$ |
| 25 | $100 \pm 8$ | $54.3 \pm 16.7$ |
| 50 | $160 \pm 8$ | $87.3 \pm 26.3$ |
| 100 | $208 \pm 8$ | $113.2 \pm 33.9$ |
| 200 | $234 \pm 14$ | $127.1 \pm 38.6$ |

Table S2: CheY-YFP concentrations as a function of IPTG

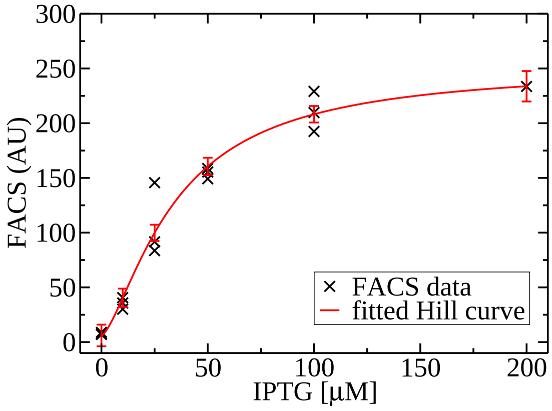


Figure S6: CheY-YFP expression levels as a function of IPTG concentration

Concentrations for typical IPTG concentrations are given in Table S2. The errors are given by the 68% confidence intervals.

In order to calculate absolute concentrations of CheY-YFP from FACS, two samples, one from a population with 10 $\mu$M and the other with 25 $\mu$M IPTG induction were measured with a fluorometer and FACS. The fluorometer was calibrated by measuring a series of solutions of known YFP concentrations. Furthermore, the number of cells per volume was determined by two different methods, i.e. plating and Neubauer chamber. We calculated for each IPTG-induction a weighted mean for the cell density and estimated the error based on the observed range of measured values. In this way we obtained for the molecule numbers per cell

$$10\ \mu\text{M IPTG} : \#\text{YFP\_molecules/cell} = (1.27 \pm 0.22) \times 10^4 \tag{92}$$

$$25\ \mu\text{M IPTG} : \#\text{YFP\_molecules/cell} = (3.16 \pm 0.57) \times 10^4 \tag{93}$$

and from this and the corresponding measured FACS values the scaling factors

$$10\ \mu\text{M IPTG} : \#\text{YFP\_molecules/cell} = (281 \pm 48) \times \text{YFP\_facs} \tag{94}$$

$$25\ \mu\text{M IPTG} : \#\text{YFP\_molecules/cell} = (323 \pm 58) \times \text{YFP\_facs}. \tag{95}$$

This gives us therefore a mean scaling factor

$$\#\text{YFP\_molecules/cell} = (302 \pm 38) \times \text{YFP\_facs} \tag{96}$$

Assuming the same expression ratio of $0.39 \pm 0.02$ between *cheY-yfp* and *cheZ-cfp* as for genomic *cheY* and *cheZ* (Li & Hazelbauer (2004)), we get

$$\#\text{CFP\_molecules/cell} = (118 \pm 16) \times \text{YFP\_facs}. \tag{97}$$

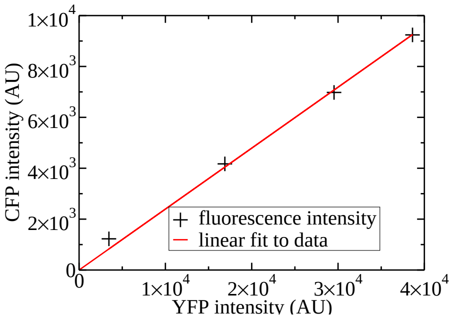


Figure S7: Correlated Expression of CheY-YFP and CheZ-CFP in swarm assay

With a cellular volume of $V_c \approx (0.92 \pm 0.25)$ fl concentration values in units of $\mu$M are given by

$$[\text{YFP}] = (0.54 \pm 0.17) \times \text{YFP\_facs} \tag{98}$$
$$[\text{CFP}] = (0.21 \pm 0.07) \times \text{YFP\_facs}. \tag{99}$$

## 7.2 Calibration of Imaging Scales

Another method to measure expression levels is given by analyzing microscopy images. This methods yields single cell data and is important for quantifying expression levels in swarm assays, where bacteria are placed on an agar medium in a petri-dish and are allowed to perform chemotaxis for several hours. Depending on the chemotaxis efficiencies of the bacteria strains, a characteristic pattern emerges: bacteria with low chemotaxis efficiency reside in the middle of the petri dish, whereas the better performing phenotypes are found in an outer ring.

In order to calibrate the fluorescence scales for YFP and CFP, we have to assume, that the bacteria in the inner region are equivalent to those grown in liquid medium. The latter ones are used for FACS, Fluorometer and FRET-experiments. At first we check whether the linear scaling for CFP- and YFP also holds in this case. Calculating the mean of the YFP- and CFP-fluorescence for different IPTG-levels shows, that linear scaling applies as shown in Fig. 6. We further assume, that the expression levels are a similar function of the IPTG-concentration as given by Eq. (91). However, fluorescent intensities measured by imaging differ by a scaling factor $\alpha_I$,

$$\text{imaging} = \alpha_I \left( p_1 \frac{\text{IPTG}^{p_4}}{\text{IPTG}^{p_4} + p_2^{p_4}} + p_3 \right), \tag{100}$$

which is determined by fitting this expression to the data obtained from swarm assays, using only $\alpha_I$ as a free parameter. The remaining parameter values are the same as given in Eq. (91). The resulting curve for YFP is shown in Fig. 7. The obtained scaling factors are given by $\alpha_I^{\text{YFP}} = 263 \pm 29$ and $\alpha_I^{\text{CFP}} = 63 \pm 8$.

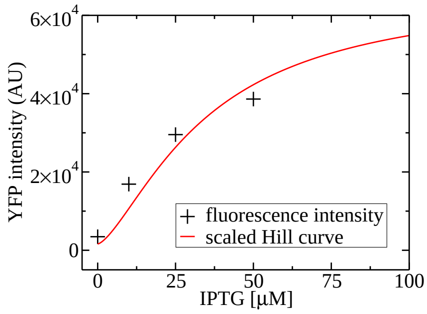


Figure S8: Mean CheY-YFP intensity as a function of IPTG

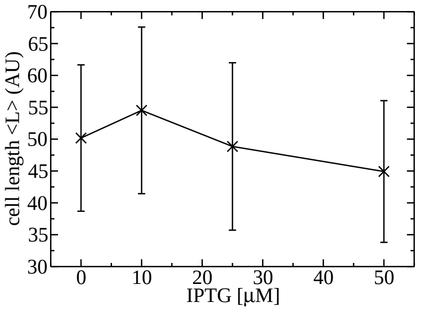


Figure S9: Mean length of bacteria as a function of IPTG

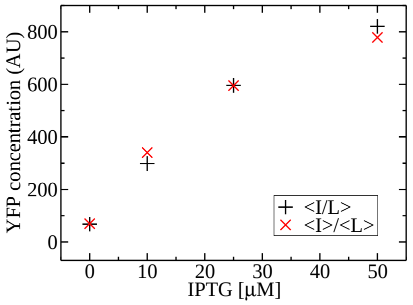


Figure S10: $< I/L >$ vs. $< I > / < L >$ as a function of IPTG

Fluorescent intensity $I$ is a measure for molecule numbers per cell, whether measured by FACS, fluorometer or microscope. However, biologically more important are molecular concentrations. This was accounted for by determining also the length $L$ of bacteria, which allowed us to calculate a concentration measure, $I/L$. Until now we assumed, that it is valid to use $\langle I/L\rangle = \langle I\rangle / \langle L\rangle$. This however does not have to be true, since $L$ and $I$ could be correlated. First of all, $L$ does not seem to be a function of the used IPTG concentrations, as depicted in Fig. 8. The mean length is given by $\langle L\rangle = 50 \pm 13$, which was calculated by using all data. Plotting $\langle I/L\rangle$ and $\langle I\rangle / \langle L\rangle$ as a function of the IPTG concentrations give similar results, see Fig. 9. Hence, it seems to be valid to assume $\langle I/L\rangle = \langle I\rangle / \langle L\rangle$.

This allows us to determine the "length concentrations" $\langle I/L\rangle$ as a function of IPTG, yielding

$$[\mathrm{YFP}]_L = (5.3 \pm 1.5) \times \mathrm{YFP\_facs} \tag{101}$$

$$[\mathrm{CFP}]_L = (1.3 \pm 0.4) \times \mathrm{YFP\_facs}. \tag{102}$$

Finally, we want to calculate the concentrations in $\mu$M, using (98) and (99), yielding

$$[\mathrm{YFP}] = (0.10 \pm 0.05) \times [\mathrm{YFP}]_L \tag{103}$$

$$[\mathrm{CFP}] = (0.17 \pm 0.07) \times [\mathrm{CFP}]_L. \tag{104}$$

This can now be used to calibrate the imaging scales in units of $\mu$M, but the estimated error seems to be too small, since it does not take into account the uncertainty of determining the functional dependence of FACS on IPTG.

## References


[1] Keymer, J. E., Endres, R. G., Skoge, M., Meir, Y., & Wingreen, N. S. (2006) *Proc. Natl. Acad. Sci. U S A* **103**(6), 1786–91.

[2] Shimizu, Thomas S, Tu, Yuhai, & Berg, Howard C June (2010) *Molecular Systems Biology* **6**, 382.

[3] Li, M. & Hazelbauer, G. L. (2004) *J. Bacteriol.* **186**, 3687–3694.

[4] Anand, GS. & Stock, AM. (2002) *Biochemistry* **41**(21), 6752–60.

[5] Turner, L., Ryu, W. S., & Berg, H. C. (2000) *J Bacteriol* **182**(10), 2793–801.

[6] Berg, H. C. & Brown, D. A. (1972) *Nature* **239**(5374), 500–4.

[7] Cluzel, P., Surette, M., & Leibler, S. (2000) *Science* **287**, 1652–1655.

[8] Block, S. M., Segall, J. E., & Berg, H. C. (1983) *J Bacteriol* **154**(1), 312–23.

[9] Segall, J. E., Block, S. M., & Berg, H. C. (1986) *Proc. Natl. Acad. Sci. U S A* **83**(23), 8987–91.

[10] Korobkova, E., Emonet, T., Vilar, J. M. G., Shimizu, T. S., & Cluzel, P. (2004) *Nature* **428**, 574–578.

[11] Mello, B. & Tu, Y. (2003) *Biophys. J.* **84**, 2943–2956.

[12] Alon, U., Surette, M. G., Barkai, N., & Leibler, S. (1999) *Nature* **397**, 168–171.

[13] Kentner, D. & Sourjik, V. (2009) *Mol Syst Biol* **5**, 238.
